# Conflictive Uncertainty: A Framework for Understanding the Aversion to Conflicting Information in Social Contexts

**DOI:** 10.1177/01461672251386102

**Published:** 2025-11-20

**Authors:** Guangyu Zhu, Yiyun Shou, Michael Smithson, Michael J. Platow

**Affiliations:** 1 School of Medicine and Psychology, The Australian National University, Canberra, Australian Capital Territory, Australia; 2 Saw Swee Hock School of Public Health, National University of Singapore and National University Health System, Singapore; 3 Lloyd’s Register Foundation Institute for the Public Understanding of Risk, National University of Singapore, Singapore

**Keywords:** conflicting information, conflictive uncertainty, ambiguity, source credibility

## Abstract

Conflicting information can significantly undermine emotions, cognition, and behavior. This paper aims to understand the negative impact of conflicting information through the lens of conflictive uncertainty. Conflictive uncertainty encompasses two dimensions: the epistemological dimension, which involves uncertainty and ambiguity about outcomes and probabilities, and the interpersonal dimension, which arises from doubts about the credibility of sources. Three experiments were conducted to test this framework. Experiment 1 found that, under conflictive uncertainty, participants rated lower source credibility and exhibited weaker preferences compared to ambiguity. Experiment 2 revealed that the negative impact of conflicting information on the strength of preference was mediated by reduced source credibility and increased perceived uncertainty. Experiment 3 demonstrated that neutralizing the loss of credibility mitigated the adverse effects of conflicting information on the strength of preference. These findings highlight the roles of source credibility and perceived uncertainty in understanding the negative effects of conflicting information on decision-making.

## Introduction

The prevalence of conflicting information, particularly in the health domain, has attracted significant attention in the research community (Carpenter et al., 2016; Hämeen-Anttila et al., 2014; Nagler & LoRusso, 2017; Nagler et al., 2020). A relevant case is vaccine safety. During the COVID-19 pandemic, while some sources emphasized a strong scientific consensus on vaccine efficacy, others highlighted rare adverse events. Such conflicting messages from seemingly credible sources can create confusion, reduce public trust, and induce skepticism toward vaccination (Gehrau et al., 2021; Giubilini et al., 2023). Conflicting information refers to two or more propositions that are logically inconsistent with one another (Carpenter et al., 2016). Such conflicts are common in areas such as vaccination (Ahn & Kahlor, 2023; Nagler et al., 2020), mammography screening (Nagler et al., 2019, 2022), nutrition (Lee et al., 2018; Nagler et al., 2022), dietary fats and carbohydrates (Clark et al., 2019), cancer prevention (Iles et al., 2022), medication recommendations (Carpenter et al., 2010, 2014), electronic cigarette use (Tan et al., 2017), and scientific discovery (Nagler et al., 2023). According to the Annenberg National Health Communication Survey, 72% of U.S. adults report medium to high exposure to conflicting nutrition information (Nagler, 2013). Similarly, several studies have found that over 50% of participants have encountered conflicting information on health-related topics (Hämeen-Anttila et al., 2014; Niederdeppe & Levy, 2007; Taplin et al., 1997), underscoring the prevalence of such conflicts in everyday life.

The prevalence of conflicting information can have negative impacts on individuals, influencing their emotions, cognitions, and behaviors. Research shows that exposure to conflicting information is associated with increased negative emotions, such as annoyance, frustration, and distress (Nagler et al., 2019). It also results in adverse cognitive outcomes, including confusion (Lee et al., 2017; Nagler et al., 2022), uncertainty or ambiguity (Han et al., 2009; Li et al., 2020; Nagler et al., 2021, 2023), distrust of information sources (Ding et al., 2011; Leiserowitz et al., 2013; Nagler et al., 2022; Shi et al., 2023), and indecision (Adriatico et al., 2022; Carpenter et al., 2016). In addition, conflicting information can discourage individuals from taking action, leading to decreased medication adherence, lower intentions to engage in health-promoting behaviors, and reduced willingness to participate in cancer prevention practices (Carpenter et al., 2014; Kobayashi, 2018; Marshall & Comello, 2019).

Despite its significance, the effects of conflicting information have not been adequately addressed, possibly due to the absence of a unified framework. The outcomes related with conflicting information span multiple levels—individual and societal—and various domains, including emotions, cognitions, and behaviors. Addressing these outcomes is crucial to mitigating the threat posed by conflicting information to the overall society’s well-being (Carpenter et al., 2016). Thus, an interdisciplinary framework is essential to disentangle the causal relationships between different outcomes effectively.

### A Framework of Conflictive Uncertainty

The impact of conflicting information on individuals should not be viewed as independent, distinct phenomena, but rather as manifestations of different stages of maladaptation to conflictive uncertainty. Conflictive uncertainty refers to “uncertainty arising from disagreement about states of reality that the receiver believes cannot be true simultaneously” (Smithson, 2022, p. 2). The conflictive uncertainty comprises two layers. The first layer of uncertainty is epistemological, stemming from ignorance about the true state of the world. This can be linked to literature on ambiguity and ambiguity aversion (Ellsberg, 1961; Hsu & Camerer, 2004; Machina & Siniscalchi, 2014). The second layer centers around the credibility of information sources, associated with research on source credibility (Hovland et al., 1953).

As for the epistemological layer, conflicting information often presents multiple possible states of the world, creating a sense of ambiguity. Ambiguity is defined as “a particular type of uncertainty due to the nature of one’s information concerning the relative likelihood of events” (Ellsberg, 1961, p. 657). While Ellsberg argued that ambiguity is a subjective experience, it can be objectively identified in situations involving unreliable or highly conflicting information. Studies on conflicting health information have shown that exposure to conflicting information increases perceived ambiguity (Nagler, 2013; Nagler et al., 2022). Han et al. (2018) and Gillman et al. (2023) also found that perceived uncertainty about vaccination and vaccination hesitancy were associated with individuals’ ambiguity attitudes. Meanwhile, the literature on ambiguity aversion suggests that people tend to avoid uncertain outcomes, especially those with unknown probabilities (Kocher et al., 2018; Machina & Siniscalchi, 2014).

Regarding the interpersonal layer, earlier studies have shown that conflictive uncertainty is more aversive than ambiguity, termed as conflict aversion (Cabantous, 2007; Cabantous et al., 2011; Pushkarskaya et al., 2010, 2015; Smithson, 1999; Smithson et al., 2019). Some findings suggest that conflictive uncertainty might go beyond ignorance about outcomes and their likelihoods and can stem from doubts about the credibility of the sources (Hu & Shyam Sundar, 2010; Jensen & Hurley, 2012; Smithson, 1999; Visschers, 2017). For example, Smithson (1999) found that participants perceive sources providing conflicting information as less credible than those offering ambiguous information. The decreased source credibility associated with conflicting information can be attributed to the consistency heuristic that individuals use when evaluating the credibility of information and its sources (Gugerty & Link, 2020). People who adopt the consistency heuristic judge the credibility of information and its sources based on how internally consistent they are and how consistent they are with other information or sources. The underlying reasoning is that there is only one truth about a given event; therefore, if two sources offer conflicting judgments, at least one or possibly both must be wrong. Neural imaging studies have also revealed that ambiguity and conflictive uncertainty are related to distinct brain regions (Bhanji & Delgado, 2014; Euston et al., 2012; Pushkarskaya et al., 2010). Ambiguity is linked to the medial prefrontal cortex, a brain region involved in decision-making, while conflictive uncertainty activates the ventral striatum, which is associated with social interaction. These findings suggest that processing conflict information is not only about making decisions with limited information, but also associated with processing social cues related to the source of the information. These two layers of conflictive uncertainty together contribute to people’s perceived uncertainty about conflicting information as well as the decreased behavioral intentions associated with conflicting information.

### Addressing Conflictive Uncertainty

The epistemological layer of conflictive uncertainty is often challenging to resolve, as it stems from limitations in knowledge and information (Gillman et al., 2023; Han et al., 2018; Nagler, 2013, 2022). For example, in the context of vaccination effectiveness, the epistemological layer of conflictive uncertainty can manifest when scientific studies provide varying predictions about the incidence of side effects after vaccination among the population. These inconsistent predictions are not merely a matter of differing opinions but arise from inherent limitations in data availability, modeling techniques, and the complexity of the human immune system. Resolving such uncertainties requires methodology improvement, more comprehensive data, and collaborative interdisciplinary approaches, which may take a significant amount of time before a full resolution.

In contrast, the interpersonal layer of conflictive uncertainty can be more manageable. According to the literature on source credibility, two key factors shape perceptions of credibility: trustworthiness and expertise. Trustworthiness refers to the audience’s confidence in the source’s intent to provide honest, sincere, and unbiased information, while expertise relates to the audience’s perception of the source’s knowledge or skills on the topic (Hovland et al., 1953). When faced with conflicting information, individuals may develop suspicions about the trustworthiness of the sources, attributing the conflict to potential bias or undisclosed personal interests (Dieckmann et al., 2017). Alternatively, they may question the competence and expertise of the sources, a phenomenon evident in growing skepticism toward the authenticity of media and the scientific community in response to conflicting information (Dieckmann et al., 2017; Leiserowitz et al., 2013; Nagler et al., 2022; Shi et al., 2023). This skepticism can lead to disengagement from these sources (Koehler & Gershoff, 2003; Liu & Chang, 2017), amplifying the negative consequences associated with conflicting information. Thus, addressing the interpersonal dimension of conflictive uncertainty can be achieved through strategies that alleviate suspicion about the source’s trustworthiness and expertise.

### The Current Research

Conflictive uncertainty consists of both an epistemological layer (linked to ambiguity and ignorance about the true state of the world) and an interpersonal layer (linked to distrust and reduced credibility in the information source). As compared to ambiguous information, conflicting information is more directly associated with reduced source credibility due to explicit contradictions between sources. Such a difference in source credibility further shapes individuals’ perceived uncertainty in context, which in turn influences their preferences for options associated with such information. This study focuses on the interpersonal layer of conflictive uncertainty and aims to develop an intervention to mitigate the negative outcomes associated with conflicting information.

This study uses ambiguous information as a control condition and compares it with conflicting information to isolate the interpersonal layer of conflictive uncertainty. Both the ambiguous and conflicting information conditions present the same range of outcome values or probabilities but differ in the level of agreement between the two pieces of information (nonconflictive vs. conflictive). Theoretically, the ambiguous information condition simulates epistemological uncertainty resulting from a lack of knowledge, while the conflicting condition introduces interpersonal uncertainty through disagreement between sources. In practice, however, ambiguous information may also carry some degree of loss in credibility, particularly when its vagueness or incompleteness is attributed to the source’s lack of expertise or transparency (Han et al., 2009, 2018; Metzger et al., 2010). Therefore, this study focuses on the relative credibility of sources under ambiguous versus conflicting information conditions, rather than assuming that ambiguity entails no credibility loss. Prior research has also shown that ambiguous information can lower credibility judgments when compared to a single, coherent message (Visschers, 2017). As such, both conditions allow room for improving perceived source credibility, supporting the use of a credibility-enhancing intervention across both types of uncertainty.

Two key cognitive components, perceived source credibility and perceived uncertainty, are identified from prior analyses, while the behavioral outcome is boiled down to a single, unified construct, “strength of preference” (Alós-Ferrer, & Garagnani, 2021; Cavagnaro & Regenwetter, 2023; Köbberling, 2006), which refers to the intensity or degree to which an individual favors one option over another. According to this framework, the causal relationships between the components are assumed as follows: the type of information influences the perceived credibility of its source, which in turn shapes the level of uncertainty individuals experience, ultimately determining the strength of their preferences. Therefore, based on Pearl’s (2009) three ladders of causation (association, intervention, counterfactual), it was hypothesized that:

**Association:**
Compared to an ambiguous information condition, participants will express lower source credibility (H1), higher perceived uncertainty (H2), and lower strength of preference (H3) under the conflicting information condition.
**Intervention:**
Compared to a control condition, an intervention that increases (decreases) perceived source credibility will decrease (increase) perceived uncertainty (H4) and increase (decrease) strength of preference (H5) under both types of information conditions.
**Counterfactual:**
Restoring perceived source credibility under the conflicting information condition to the level of the ambiguous information condition will eliminate the difference in strength of preference (H6).

The experiments in this paper also establish a sequential mediation model involving the type of information, perceived source credibility, perceived uncertainty, and strength of preference to support the framework. Moreover, given that a previous study found that a minority of participants did not rate the source credibility of conflicting information lower than that of ambiguous information (Smithson, 1999), this study also explored potential causal heterogeneity within the mediation relationship.

## Experiment 1

This experiment investigated differences in perceived source credibility and strength of preference between the ambiguous and conflicting information conditions across four scenarios: health, gambling, investment, and marketing. These scenarios are widely used in decision-making studies (Ellsberg, 1961; Smithson, 1999; Smithson & Campbell, 2009; Weyerer et al., 2019). In addition, the experiment explored the mediation relationship between the type of information, perceived source credibility, and strength of preference.

### Method

#### Participants and Design

A total of 100 English-speaking adults from the United States and the United Kingdom participated in this experiment. This experiment is a mixed design with type of information (ambiguous vs. conflicting) as the between-subject variable and type of scenario (health, gambling, investment, marketing) as the within-subject variable. Based on the G*power 3.1 (Faul et al., 2009), the sample size of 100 was sufficient to detect a medium main effect (*f*^2^ = .25) of information type on strength of preference^1^ with .05 significance level and .90 power in repeated measures ANOVA with an assumed medium correlation among repeated measures (*r* = .50). Based on Monte Carlo Power Analysis for Indirect Effects (Schoemann et al., 2017), the sample size of 100 was also sufficient to detect a medium size mediation relationship (X-M: a = .39, M-Y: b = .39, X-M-Y: ab = .15) with .90 power (Sim et al., 2022).

The participants had a mean age of 37.66 (*SD* = 13.23), with 59% of participants being male and 41% being female. Participants included 82% Caucasian adults, 4% African, 9% Asian, and 5% people with other racial backgrounds. Participants were recruited through the online platform Prolific. They were paid £0.75 (around US$0.95) for their participation.

All data, analysis code, and research materials are available at https://osf.io/tcv63. Data were analyzed using R, version 4.3.1. The preregistration of this experiment can be found on OSF: https://osf.io/mhvue. Hypotheses 1 and 3 were preregistered. The ethical aspects of the studies have been approved by the ANU Human Research Ethics Committee (Protocol 2021/395).

#### Measures

##### Scenario Description

In the experiment, participants were asked to imagine making decisions in four hypothetical scenarios: health, gambling, investment, and marketing. Within each scenario, they were presented with probability information^2^ predicting the outcomes of each decision from two online sources. These sources were set to be online bloggers to maintain cross-domain consistency and applicability, as well as to enable subsequent manipulation of the sources’ occupations. The information was either ambiguous or conflicting.

The formal task included two conditions: ambiguous information and conflicting information. Under the ambiguous information condition, X% represented an imprecise but consistent probability estimation, such as 30% to 70% for both sources. Under the conflicting information condition, X% represented a conflicting but precise probability estimation, with one source providing a probability of 30% and the other 70%. This presentation of ambiguous and conflicting information can be linked to many ambiguity aversion studies such as Cabantous (2007) and Aggarwal and Mohanty (2022). The probabilities were set at 30% and 70% to avoid ambiguity-seeking behavior typically observed at lower likelihoods. The descriptions of the scenarios are as follows:

Health: Suppose you are considering the purchase of an over-the-counter medication, Medicine A. This medicine is supposed to help cure a common respiratory disease and has no side effects in humans. However, because Medicine A is a newly developed medicine, its effectiveness has not been fully explored. There are two online bloggers saying that they have conducted the pilot studies with some people and providing their judgment about the effectiveness of this medicine:

**Table:** 

*Medicine A*	
*Jacob*	*X% of disease sufferers using Medicine A show improvement*
*William*	*X% of disease sufferers using Medicine A show improvement*

Gamble: Suppose you are considering taking a gamble, Gamble A. This gambling game will reward you with $100 when you win and nothing when you lose. The winning probability of the gamble depends on some random events (like a coin toss). There are two online bloggers saying they have taken a similar gamble before and providing their judgment about the winning probabilities of Gamble A.

**Table:** 

*Gamble A*	
*John*	*There is an X% chance of winning $100*
*Ethan*	*There is an X% chance of winning $100*

Investment: Suppose you are considering investing in a stock, Stock A. Stock A is issued by a newly listed company, so it is currently uncertain about its future trends. There are two online bloggers saying that they have investigated the company that issued this stock and providing their estimation of the probability of a positive return of this stock.

**Table:** 

*Stock A*	
*Matthew*	*There is an X% chance that this stock will have a positive return*
*James*	*There is an X% chance that this stock will have a positive return*

Marketing: Suppose you are considering buying new headphones, Headphone A. This headphone was newly released and there are few reviews on its quality and usefulness. There are two online bloggers saying that they have conducted trials with some people and providing their judgment of the percentage of people who like these headphones.

**Table:** 

*Headphone A*	
*Michael*	*X% of the customers like this headphone*
*Ryan*	*X% of the customers like this headphone*

##### Trust Propensity

Trust propensity reflects an individual’s tendency to trust strangers (Heyns & Rothmann, 2015). People with higher trust propensity are more likely to rate source credibility higher (Lucassen & Schraagen, 2012), but they are also more tolerant of uncertainty, and more willing to accept options with uncertain outcomes (Colquitt et al., 2007; Gillman et al., 2023; Thielmann & Hilbig, 2015). Consequently, trust propensity could confound the relationship between perceived source credibility and strength of preference for uncertain options and should be controlled. Trust propensity was measured using the General Trust Scale (Yamagishi & Yamagishi, 1994), where participants rated each item on a scale from 1 (“strongly disagree”) to 5 (“strongly agree”). An example item is, “Most people tell a lie when they can benefit by doing so.” The General Trust Scale has shown good validity and reliability in previous research (Jasielska et al., 2021). In this experiment, the alpha coefficient for the scale was .86 (95% CI = [.82, .90]).

##### Perceived Source Credibility

Perceived source credibility was measured using a slider scale ranging from 0 (“not credible at all”) to 100 (“very credible”) in response to the question: “How credible do you think the sources are?” The slider scale, with its wider range of values, provided a more fine-grained measurement of participants’ responses compared to the Likert-type scale (Roster et al., 2015). Although credibility is often considered a multidimensional construct encompassing factors such as trustworthiness and expertise, results from a pilot study revealed that participants’ responses to different credibility-related questions were highly correlated (*r* > .75). This finding suggests that lay participants may not clearly distinguish between terms such as “trustworthy” and “competent” (see Appendix A).

##### Strength of Preference

Strength of preference is commonly measured through tasks like willingness to pay/sell (Breidert et al., 2006) or certainty equivalents (Krahnen et al., 1997). However, these methods are typically limited to scenarios involving monetary or quantitative outcomes, while conflicting information often arises in contexts beyond economic judgments, such as medical settings. To address this limitation, the matching probability task was introduced as a cross-domain measure of the strength of preference. Matching probability represents the precise probability a person is willing to accept in exchange for an uncertain probability. Initially introduced by Dimmock et al. (2016) to measure individuals’ strength of preference under ambiguity, the matching probability task was validated by Baillon et al. (2018), who demonstrated its reliability in capturing strength of preference between options.

The procedure for the matching probability task is similar to the certainty equivalent task. Participants indicate their preferences between a reference option with a precise probability (e.g., 50%, which adjusts based on their choices) and a target option with an uncertain probability (e.g., 30–70%). For each comparison, participants can choose “Prefer Target Option (a),” “Prefer Reference Option (b),” or “No Preference.” The precise probability in the reference option adjusts iteratively based on participants’ responses: if participants prefer the reference option, the precise probability decreases to 25% (the midpoint between 50% and 0%); if they prefer the target option, it increases to 75% (the midpoint between 50% and 100%).

This process (illustrated in Figure 1) continues until participants either express no preference between the two options or switch their preference within a 5% interval (e.g., preferring the reference option at 45% but the target option at 40%). If participants express no preference, the precise probability at that point is recorded as the matching probability for the target option. If participants switch their preference within a 5% interval, the midpoint of that interval is taken as the matching probability (e.g., 42.5% in the above-mentioned example).

**Figure 1.**
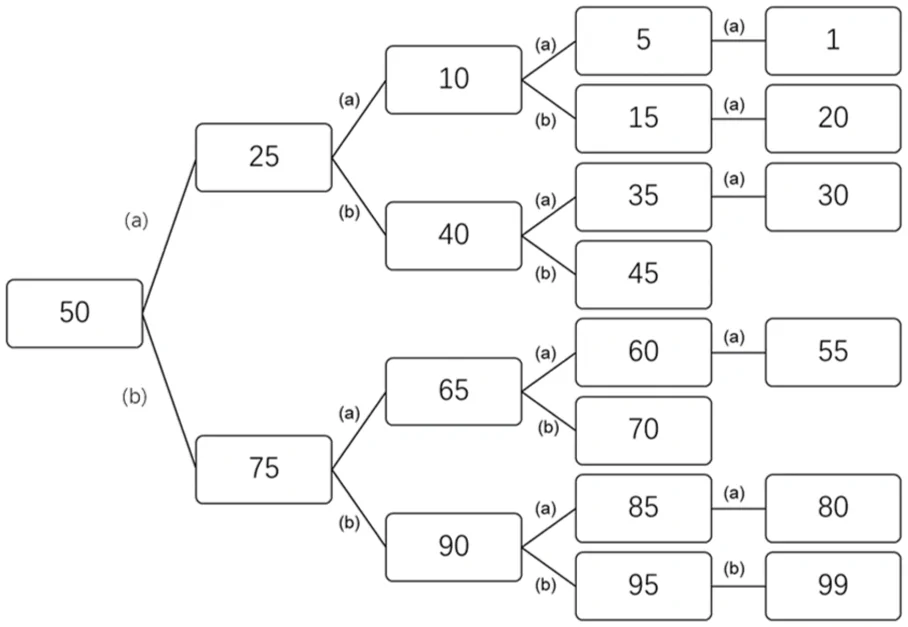
The Procedure of the Multiple Pairwise Comparisons.

#### Procedure

Participants from the Prolific platform were invited to complete an online survey hosted on the Qualtrics platform. Informed consent was obtained before they began the survey. The experiment started with questions assessing trust propensity, which were presented at the beginning to prevent potential interference from the experimental manipulation on self-reported trust tendencies. Participants then proceeded to the formal part of the study, where they read information from different sources and answered questions about perceived source credibility and strength of preference. Each participant completed four scenarios sequentially, assigned to either the ambiguous or conflicting information condition. The order of sources was counterbalanced, and the order of the scenarios was randomized for each participant. After completing all tasks, participants answered demographic questions and submitted their responses.

### Results

The descriptive statistics of participants’ trust propensity, perceived source credibility, and strength of preference are summarized in Table 1. This table also includes the effect size (Cohen’s *d*) of the difference in these variables between the ambiguous and conflicting information conditions.

**Table 1:** Descriptive Information of the Variables

	Ambiguous information			Conflicting information			Cohen’s *d*
Variables	*Mean*	*Median*	*SD*	*Mean*	*Median*	*SD*	
TP	3.46	3.67	.74	3.40	3.42	.66	.08
Health							
SC	38.70	41.50	24.80	20.56	19.50	17.63	.84
SP	43.92%	47.50%	11.37	34.24%	40.00%	18.19	.64
Gambling							
SC	38.10	36.50	24.88	20.50	20.00	16.54	.83
SP	44.95%	47.50%	12.17	36.72%	41.25%	15.77	.58
Investment							
SC	39.18	37.50	23.39	23.08	20.00	16.65	.79
SP	44.70%	47.50%	10.41	38.12%	43.75%	16.06	.49
Marketing							
SC	49.08	51.00	22.41	31.50	30.00	19.66	.83
SP	47.10%	47.50%	9.55	41.72%	47.50%	15.47	.42

*Note*: TP = Trust Propensity; SC = Source Credibility; SP = Strength of Preference.

#### Pretest Trust Propensity Check

An independent t-test found that there was no significant difference in the trust propensity between the ambiguous information condition (*M* = 3.46, *SD* = .74) and the conflicting information condition (*M* = 3.40, *SD* = .66), *t*(98) = .41, *p* = .685, indicating that participants under both conditions exhibited nearly identical levels of trust tendency.

#### Perceived Source Credibility

A mixed effect model (lme4 package; Bates et al., 2015) was conducted on perceived source credibility, with type of information and type of scenario as fixed effects, and including a random intercept for participants. The results showed a significant main effect of type of information on perceived source credibility, *F*(1, 98) = 20.38, *p* < .001, η^2^ = .172. Participants reported higher perceived credibility for sources providing ambiguous information (*M* = 41.27, *SD* = 24.14) than those providing conflicting information (*M* = 24.43, *SD* = 18.02), supporting Hypothesis 1. The type of scenario had a significant main effect on perceived source credibility, *F*(3, 264) = 19.93, *p* < .001, η^2^ = .169. The post hoc Tukey contrasts test using the Holm method of adjusted *p* values (the multcomp package; Hothorn et al., 2016) revealed that participants reported the highest perceived credibility for sources in the marketing scenario compared to each of the other three scenarios (investment: *z* = -4.42, *p* < .001; health: *z* = -4.63, *p* <.001; gambling: *z* = -4.90, *p* <.001). The interaction between type of information and type of scenario was not significant (*p* = .206).

#### Strength of Preference

A mixed-effect model was also conducted to analyze participants’ strength of preference for uncertain options. The results found a significant main effect of type of information on strength of preference, *F*(1, 98) = 10.87, *p* =.001, η^2^ = .100. Participants demonstrated a stronger preference for options with ambiguous information (*M* = 45.17, *SD* = 10.89) than those with conflicting information (*M* = 37.70, *SD* = 16.50), supporting Hypothesis 3. Type of scenario also had a significant effect on the strength of preference, *F*(3, 294) = 5.61, *p* < .001, η^2^ = .054. The post hoc Tukey contrasts test revealed that participants demonstrated the greatest preference for options in the marketing scenario compared to the health, *z* = 4.03, *p* < .001, and gambling, *z* = 2.70, *p* = .035, scenarios, while there was no significant difference in the strength of preference for options in the marketing scenario compared to investment scenario, *z* = 2.26, *p* = .09. The interaction between the type of information and the type of scenario not significant (*p* = .389).

#### Mediation Analysis

A causal mediation analysis was conducted using the mediation package in R (Tingley et al., 2014) to examine whether the effect of information type on strength of preference was mediated by perceived source credibility. This model is based on the potential outcome framework (Rubin, 2005) and estimates the mediation relationship through a nonparametric algorithm outlined by Imai, Keele, and Tingley (2010). The results from this model align with those obtained using Sobel’s test (Sobel, 1982). However, a key advantage of this model over Sobel’s test is that it does not rely on the assumption of normality for the distribution of the product of coefficients (Imai, Keele, & Yamamoto, 2010).

Given that each participant was nested within four scenarios, the repeated measures structure violated the independence assumption underlying traditional single-level mediation models. To address this, two mixed-effects models with random intercepts for participants and scenarios were fitted as the foundation for the mediation analysis, using the lme4 package (Bates et al., 2015). The first model estimated the fixed effect of information type on source credibility, while the second model estimated the fixed effect of source credibility on strength of preference, accounting for variations across participants and scenarios. The results from the mediation analysis showed that there was a significant total effect of type of information on strength of preference (β = -.51, 95% CI = [-.82, -.21], *p* < .001), and 33.0% of the total effect could be attributed to the significant indirect effect via perceived source credibility (β = -.17, 95% CI = [-.29, -.07], *p* < .001). There was also a significant direct effect of type of information on strength of preference (β = -.34, 95% CI = [-.65, -.04], *p* = .027).

### Discussion

This experiment found that participants reported lower perceived source credibility and strength of preference for options with conflicting information compared to those with ambiguous information across marketing, gambling, investment, and medical scenarios. These findings are consistent with previous research on conflict aversion (Cabantous, 2007; Cabantous et al., 2011; Pushkarskaya et al., 2010, 2015). In addition, the mediation model provided supporting evidence that aversion to conflicting information is driven by decreased perceived source credibility.

## Experiment 2

Experiment 2 expanded the previous mediation model by introducing perceived uncertainty as a second mediator. In addition, it manipulated source credibility to further examine the causal relationships among perceived source credibility, perceived uncertainty, and strength of preference.

This experiment focused on the health scenario from Experiment 1, using the occupation of the sources (doctor/politician) as an intervention to influence perceived source credibility. Doctors are regarded as having higher expertise in health-related issues compared to online unauthorized sources or politicians (Lee et al., 2019). They are also considered a trustworthy profession, as indicated by the Ipsos Global Trustworthiness Index 2022 (Clemence & Jackson, 2022). Thus, a doctor as the source was expected to increase perceived source credibility. In contrast, politicians, who received the lowest trustworthiness ratings on the Ipsos Global Trustworthiness Index, were expected to decrease perceived credibility when acting as the source of health-related information.

Moreover, Experiment 2 aimed to explore causal heterogeneity within this mediation relationship. Causal heterogeneity, in this context, may arise when some participants do not perceive lower credibility for sources providing conflicting information compared to ambiguous information. Consequently, two potential subgroups of participants may exist: (1) those who perceive lower credibility for sources providing conflicting information and (2) those who perceive equal or higher credibility for sources providing conflicting information. Experiment 2 investigated whether heterogeneity exists in source credibility judgments and, if so, whether participants in the two subgroups exhibit different patterns in perceived uncertainty and strength of preference. In addition, it explored potential factors contributing to this causal heterogeneity.

### Method

#### Participants and Design

A total of 195 English-speaking adults from the United States and the United Kingdom participated in this experiment. The participants had a mean age of 37.04 (*SD* = 14.57). This experiment is a mixed design, using type of information as a within-subject variable and source occupation as a between-subject variable. Participants were randomly assigned to one of the occupation type conditions and went through the ambiguous and conflicting information conditions sequentially. The order in which participants were exposed to the types of information was randomized, with half receiving ambiguous information first and the other half receiving conflicting information first.^3^ Based on the G*power 3.1 (Faul et al., 2009), a sample size of 195 was sufficient to detect a medium main effect of information type on strength of preference, as well as its interaction with occupation type (*f*^2^ = .25) with .05 significance level and .95 power in repeated measures ANOVA with an assumed medium correlation among repeated measures (*r* = .50). Based on Monte Carlo Power Analysis for Indirect Effects (Schoemann et al., 2017), this sample size is sufficient for detecting a medium size sequential mediation relationship (X-M1: a = .39, M2-Y: b = .39, M1-M2: d= .39, X-M1-M2-Y: abd = .06) with .90 power.

The sample consisted of 51% males, 46% females, and 3% nonbinary gender. Participants included 79% with Caucasian background, 6% African, 7% Asian, and 8% from other racial backgrounds. Participants were recruited through the online platform Prolific and were paid £1 (around US$1.27) for their participation.

Data were analyzed using R, version 4.3.1. The preregistration can be found at https://osf.io/yk2az. A replication of Experiment 2 can also be found in the supplementary materials. Hypotheses 1, 2, 3, 4, and 5 were registered. The preregistered beta regression analysis was replaced with a mixed ANOVA to maintain consistency with previous analyses. A comparison of the results from the beta regression and the mixed ANOVA revealed no instances where one model produced significant results, while the other did not.

#### Materials

This experiment selected the health scenario from Experiment 1. The trust propensity question, perceived source credibility question, and strength of preference were the same as the health scenario in Experiment 1. There were additional cues on source occupations below each source’s name.

##### Perceived Uncertainty

Perceived uncertainty was measured by a slider question from 0 (“not certain at all”) to 100 (“very certain”). Participants were asked: “How certain do you feel about effectiveness of the medicine?,” with lower values reflecting greater perceived uncertainty

#### Procedure

The procedure of Experiment 2 was similar to Experiment 1.

### Results

The means, medians, and standard deviations of participants’ trust propensity, perceived source credibility, perceived uncertainty, and strength of preference are summarized in Table 2. This table also includes the effect size (Cohen’s *d*) of the difference in these variables between the ambiguous and conflicting information conditions.

**Table 2:** Descriptive Information of the Variables

	Ambiguous information			Conflicting information			Cohen’s *d*
Variables	*Mean*	*Median*	*SD*	*Mean*	*Median*	*SD*	
Control							
TP	3.42	3.50	.60	-	-	-	
SC	49.21	51.00	23.86	37.82	40.00	21.38	.50
PU	44.82	50.00	22.78	33.20	32.50	22.59	.51
SP	41.64%	46.25%	16.45	35.70%	40.00%	19.36	.33
Doctor							
TP	3.29	3.33	.68	-	-	-	
SC	62.23	63.00	22.08	51.17	50.00	24.25	.48
PU	49.35	50.00	23.79	37.54	40.00	22.75	.53
SP	45.65%	47.50%	15.68	41.32%	42.50%	13.52	.30
Politician							
TP	3.40	3.50	.59	-	-	-	
SC	41.36	43.50	23.76	25.81	21.00	20.00	.71
PU	37.70	42	19.70	25.77	20.00	21.97	.57
SP	35.66%	40.00%	16.71	31.23%	32.50%	15.68	.27

Note: TP = Trust Propensity; SC = Source Credibility; PU = Perceived Uncertainty; SP = Strength of Preference; PU variable was reverse-coded, with lower scores in PU variable indicating greater perceived uncertainty.

#### Pretest Trust Propensity Check

One-way independent ANOVA found no significant difference between the control condition (*M* = 3.42, *SD* = .59), doctor condition (*M* = 3.29, *SD* = .68), and politician condition (*M* = 3.40, *SD* = .69), *F*(2, 387) = 1.48, *p* = .227, η2 =.008. This indicates that participants in these conditions exhibited a similar tendency to trust others.

#### Perceived Source Credibility

A mixed-effect model (lme4 package; Bates et al., 2015) was conducted on perceived source credibility, with type of information and source occupation as fixed effects, and including a random intercept for participants. The results found a significant main effect of information type on the perceived source credibility, *F*(1, 192) = 60.50, *p* <.001, η^2^ = .240. Participants rated the sources who provide ambiguous information (*M* = 50.97, *SD* = 24.68) as more credible than those who provide conflicting information (*M* = 38.33, *SD* = 24.17), supporting Hypothesis 1. The source occupation type also had a significant main effect on the perceived source credibility, *F*(2, 192) = 22.72, *p* < .001, η^2^ =.191. The post hoc Tukey contrasts test showed that there was a significantly lower perceived source credibility in the control condition (*M* = 43.52, *SD* = 23.28) than under the doctor condition (*M* = 56.70, *SD* = 23.76), *z* = 3.30, *p* = .002. There was also a significantly higher perceived source credibility in the control condition (*M* = 43.52, *SD* = 23.28) than the politician condition (*M* = 33.59, *SD* = 23.22), *z* = 1.98, *p* = .048. The interaction between the type of information and source occupation type was not significant (*p* = .460).

#### Perceived Uncertainty

The mixed-effect model found a significant main effect of type of information on perceived uncertainty, *F*(1, 192) = 62.62, *p* < .001, η^2^ = .251. Participants reported a lower level of uncertainty under the ambiguous information condition (*M* = 44.03, *SD* = 22.57) than under the conflicting information condition (*M* = 32.20, *SD* = 22.84), supporting Hypothesis 2. The source occupation type had a significant main effect on the perceived uncertainty, *F*(2, 192) = 5.80, *p* = .004, η^2^ =.063, supporting Hypothesis 4. A post hoc Tukey contrasts test showed that there was a significantly lower perceived uncertainty under the doctor condition (*M* = 43.45, *SD* = 23.93) than under the politician condition (*M* = 31.73, *SD* = 21.63), *z* = 2.97, *p* = .009. The interaction between the type of information and source occupation type was not significant (*p* = .998).

#### Strength of Preference

The mixed-effect model found a significant main effect of type of information on the strength of preference, *F*(1, 192) = 36.29, *p* < .001, η^2^ = .159. Participants demonstrated a stronger strength of preference for options with the ambiguous information (*M* = 41.02, *SD* = 16.70) than those with the conflicting information (*M* = 36.11, *SD* = 16.81), supporting Hypothesis 3. The source occupation type also had a significant main effect on the strength of preference, *F*(2, 192) = 6.94, *p* <.001, η^2^ =.067, supporting Hypothesis 5. A post hoc Tukey contrasts test showed that there was a significantly higher strength of preference in the doctor condition (*M* = 43.49, *SD* = 14.74) than in the politician condition (*M* = 33.45, *SD* = 16.29), *z* = 3.47, *p* = .002. The interaction between uncertainty type and source occupation type was not significant (*p* = .664).

#### Sequential Mediation Analysis

According to the theoretical analysis, the type of information influences the source credibility, followed by the perceived uncertainty, which in turn influences the strength of preference. Therefore, a sequential mediation model was fitted to estimate this ordered indirect pathway using the lavaan package (Rosseel et al., 2017). The results from the sequential mediation models were illustrated in Figure 2. The whole model showed a good fit, χ²(1) = 6.01, *p* = .014, CFI = .986, RMSEA = .113, SRMR = .025. There was a significant total effect of type of information on strength of preference, β = -4.24, 95% CI = [-7.51, -0.97], *p* = .011. There was also a significant indirect effect (X-M_1_-M_2_-Y) from type of information, via perceived source credibility, perceived uncertainty, to strength of preference, β = -1.00, 95% CI = [-1.73, -0.27], *p* = .007. There was a significant indirect effect (X-M_1_-Y) from type of information via perceived source credibility to strength of preference, β = -1.49, 95% CI = [-2.68, -0.30], *p* = .014. However, there was a nonsignificant indirect effect (X-M2-Y) from the type of information via perceived uncertainty to strength of preference, β = -.66, 95% CI = [-1.39, 0.06], *p* = .070.

**Figure 2.**
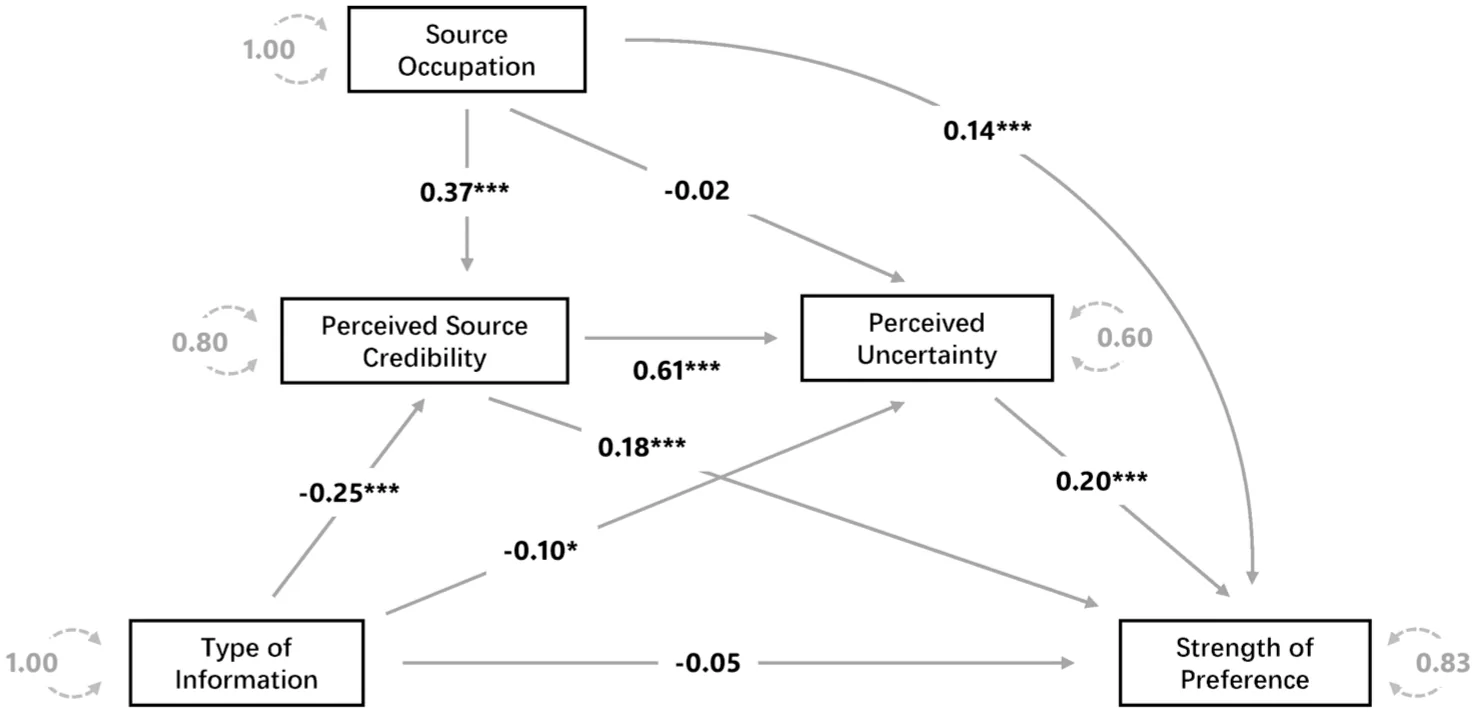
Sequential Mediation Models Results. *: significance at 0.05 level, **: significance at 0.01 level; ***: significance at 0.001 level.

#### Causal Heterogeneity Analysis

Participants’ perceived source credibility ratings in the conflicting information condition were compared to those in the ambiguous information condition. The results revealed that 36% of participants rated sources providing conflicting information as having equal or higher credibility than those providing ambiguous information, indicating an absence of interpersonal layer uncertainty among those participants. These participants were categorized under a new variable, “subgroup,” with the label “no decrease in perceived source credibility.” The remaining participants, who rated sources providing conflicting information as less credible than those providing ambiguous information, were labeled as the “decreased perceived source credibility” group.

A multigroup structural equation model (SEM) using the lavaan package (Rosseel et al., 2017) was conducted to examine whether the hypothesized sequential mediation model differed between groups. The results show that the unconstrained model, allowing subgroup differences in the direct and indirect pathway coefficients, did not significantly differ from the constrained model assuming no such differences, *χ²*(8) = 10.80, *p* = .213. However, a significant sequential pathway from type of information, through source credibility and perceived uncertainty, to strength of preference was found in the “decreased perceived source credibility” group, *b* = -.86, 95% CI = [-1.77, -0.17], *p* = .037. In contrast, the same pathway was not significant in the “no decrease in perceived source credibility” group, *b* = -1.65, 95% CI = [-3.70, 0.02], *p* = .086. Similarly, a significant indirect effect from type of information through source credibility to strength of preference was observed in the “decreased perceived source credibility” group, *b* = -1.55, 95% CI = [-3.24, -0.36], *p* = .034, but not in the “no decrease in perceived source credibility” group, *b* = -.92, 95% CI = [-3.39, 1.56], *p* = .455. These results suggest that the mediation pathways were only present among participants who decreased perceived source credibility. The correlation matrix among perceived source credibility, perceived uncertainty, and strength of preference was also calculated to support the causal heterogeneity analysis (see Appendix B).

Exploration of potential factors accounting for differences between the two subgroups found that the “no decrease in perceived source credibility” group exhibited significantly higher trust propensity (*M* = 3.51, *SD* = .66) than the “decreased perceived source credibility” group (*M* = 3.29, *SD* = .64), *t*(139) = 2.19, *d* = .34, *p* = .030. However, the two subgroups showed no significant difference in age, *t*(125) = 1.91, *d* = .29, *p* = .059, gender, *χ²*(3) = 3.56, *p* = .313, and education levels, *χ²*(4) = 4.73, *p* = .316.

### Discussion

This experiment extended the previous mediation model by incorporating perceived uncertainty as a second mediator. The results of the sequential mediation model highlighted the roles of decreased perceived source credibility and increased perceived uncertainty as key factors contributing to decreased strength of preference. In addition, the manipulation of perceived source credibility significantly influenced participants’ perceived uncertainty and strength of preference for options with uncertain information, establishing a causal relationship between perceived source credibility, perceived uncertainty, and strength of preference. While a significant indirect effect was observed from the type of information, mediated by perceived source credibility and perceived uncertainty, to strength of preference, the total effect was not significant. This lack of significance may be attributed to low statistical power in the total effect test (Loeys et al., 2015).

However, it also clarified the scope of this mediation relationship. Specifically, only 64% of participants who rated lower perceived source credibility for conflicting information compared to ambiguous information followed this pathway. Individual differences, such as trust propensity, may contribute to this causal heterogeneity. Prior research suggests that individuals with high trust propensity are less likely to feel exploited when relying on others in uncertain situations (Alarcon et al., 2018; Thielmann et al., 2020). Such individuals may be less suspicious of manipulation or deception, reducing their likelihood of discounting the credibility of sources when encountering conflicting information.

## Experiment 3

While findings from Experiment 2 provide evidence that alter of source credibility could lead to the corresponding changes in strength of preference, it remains unclear how the strength of preference would change if the perceived credibility of sources providing conflicting information were equalized with that of sources providing ambiguous information. According to Lewis’ (1973) definition of causal dependence, “Where *c* and *e* are two distinct possible events, *e* causally depends on *c* if and only if, *c* were to occur, *e* would occur; and if *c* were not to occur, *e* would not occur.” If the strength of preference causally depends on perceived source credibility, it can reasonably be predicted that the decreased strength of preference would weaken or disappear when the credibility of sources providing conflicting information is equalized with that of sources providing ambiguous information.

Experiment 3 was designed to test this hypothesis. In this experiment, participants were first assigned to the ambiguous information condition with no source occupation cues and then to the conflicting information condition, with an intervention (the doctor occupation) intended to “neutralize” the decrease in perceived source credibility. The order of conditions was fixed to ensure that credibility judgments in the ambiguous information condition were minimally contaminated by noise introduced by the intervention.

Previous research highlights the importance of timing in interventions. Once a source loses credibility, the process of recovery is often slow and incomplete (Lewicki & Bunker, 1996; Schweitzer et al., 2006). To examine the impact of timing on the effectiveness of interventions, this experiment included two conditions: early intervention and late intervention. In the early intervention condition, the intervention was implemented concurrently with participants’ exposure to the conflicting information. In contrast, under the late intervention condition, it was introduced after participants had already been exposed to the conflicting information and had made an initial decision.

### Method

#### Participants and Design

A total of 240 English-speaking adults from the United States and the United Kingdom participated in this experiment. This experiment employed a mixed design. The type of information (ambiguous vs. conflicting) was treated as a within-subject variable, and the timing of intervention (early intervention vs. late intervention) was treated as a between-subject variable. Participants were randomly assigned to the early or late intervention condition and completed the tasks in the ambiguous and conflicting information conditions sequentially. The order of the conditions was ambiguous information first and conflicting information second. Each condition (early intervention vs. late intervention) had 120 participants. According to the G*power 3.1 (Faul et al., 2009), a sample size of 120 is sufficient to detect a medium effect of the type of information on the strength of preference (d = .5), with .05 significance level and .95 power in a *t*-test.

The participants had a mean age of 40.17 (*SD* = 12.57), with 35% of the sample male and 65% female. Participants included 92% Caucasian, 2% African, 4% Asian, and 2% from other racial backgrounds. Participants were recruited through the online platform Prolific. They were paid £0.6 (around US$0.75) for their participation.

Data were analyzed using R, version 4.3.1. The preregistration can be found at OSF: https://osf.io/45qgj. Hypothesis 6 was registered.

#### Measures

The measures were the same as Experiment 2.

#### Procedure

Participants from the Prolific platform were invited to participate in an online experiment hosted on the Qualtrics survey platform. The experiment began with a measure of trust propensity, followed by the formal task. In the formal task, participants were randomly assigned to either the early or late intervention condition. Under both conditions, participants first completed the ambiguous information condition, where only the names of the sources were available. They then answered questions about perceived source credibility and strength of preference.

Next, participants entered the first round of the conflicting information condition. Those in the early intervention condition were informed that both sources were doctors and were provided with their names. In contrast, participants in the late intervention condition were only provided with the names of the sources. All participants rated their perceived source credibility and completed the strength of preference questions.

Subsequently, participants entered the second round of the conflicting information condition. In the late intervention condition, participants were now informed that the two sources from the previous situation were doctors and were asked to rate perceived source credibility and strength of preference again. Participants under the early intervention condition also completed these measures again to assess whether prompting them to reconsider their responses influenced their perceived source credibility and strength of preference. After completing the tasks, participants answered demographic questions and submitted their responses.

### Results

The means, medians, and standard deviations of participants’ trust propensity, perceived source credibility, and strength of preference are summarized in Table 3. Because the absence of decreased source credibility and difference in the strength of preference effects are null hypotheses, this experiment used a Bayesian t-test in addition to the traditional t-test to assess the strength of evidence supporting the null hypotheses. The Bayes factor for the null hypothesis BF_01_ was calculated using the BayesFactor package (Morey et al., 2015), with a default Cauchy prior (centered at 0, *r* = .707). In both *t*-tests, the null hypothesis H_0_ (M_1_ = M_2_) was compared with a two-sided alternative hypothesis H_1_ (M_1_≠M_2_).

**Table 3.** Descriptive Information of the Variables.

	Ambiguous information			Conflicting information (1^st^ round)			Conflicting information (2^nd^ round)		
Variables	*Mean*	*Median*	*SD*	*Mean*	*Median*	*SD*	*Mean*	*Median*	*SD*
Early intervention									
TP	3.62	3.83	.62	-	-	-	-	-	-
SC	53.89	53.50	21.57	48.73	50.00	20.27	48.37	50.00	20.44
SP	42.74%	47.50%	12.71	39.89%	40.00%	13.64	39.34%	40.00%	14.47
Late intervention									
TP	3.64	3.93	.64	-	-	-	-	-	-
SC	54.40	51.50	21.16	39.36	40.00	19.05	54.58	53.00	22.79
SP	44.70%	47.50%	15.06	37.77%	40.00%	15.83	42.41%	45.00%	14.47

*Note*: TP = Trust Propensity; SC = Source Credibility; SP = Strength of Preference.

#### Pretest Trust Propensity Check

An independent *t*-test revealed no significant difference in the trust propensity among the early intervention condition (*M* = 3.62, *SD* = .62) and the late intervention condition (*M* = 3.64, *SD* = .64), *t*(237) = -.26, *p* = .798, *d* = .19, BF_01_ = 6.86.

#### Perceived Source Credibility

The paired *t*-test was conducted to evaluate the effectiveness of the intervention in equalizing the perceived source credibility under both early and late intervention conditions. There was a significant difference between the ambiguous information condition (*M* = 53.89, *SD* = 21.57) and the conflicting information condition (*M* = 48.73, *SD* = 20.27) in the early intervention group, *t*(119) = 2.00, *p* = .047, *d* = .25, BF_01_ = 1.43. This indicates that the early intervention was insufficient to equalize perceived source credibility in the conflicting information condition to a level comparable to that in the ambiguous information condition.

However, in the late intervention group, there was no significant difference between the ambiguous information (*M* = 54.40, *SD* = 21.16) and the conflicting information conditions (*M* = 54.58, *SD* = 22.79), *t*(119) = .07, *p* = .944, *d* = .01, BF_01_ = 10.1. The Bayes factor provides moderate support for the null hypothesis, suggesting that the late intervention successfully equalized the perceived source credibility in the conflicting information condition to a similar level as the ambiguous information condition.

#### Strength of Preference

The paired *t*-test revealed a significant difference between the ambiguous information (*M* = 42.74, *SD* = 12.71) and the conflicting information conditions (*M* = 39.89, *SD* = 13.64), *t*(119) = 2.59, *p* = .011, *d* = .22, BF_01_ = .40, under the early intervention condition. The Bayes factor showed anecdotal support for the alternative hypothesis, indicating that the early intervention can weaken but fail to eliminate the decreased strength of preference.

However, there was no significant difference in the strength of preference between the ambiguous information (*M* = 44.70%, *SD* = 15.06) and the conflicting information conditions (*M* = 42.41%, *SD* = 14.47), *t*(119) = 1.66, *p* = .099, *d* = .16, BF_01_ = 2.63 under the late intervention condition. The Bayes factor slightly supports the null hypothesis, indicating that the late intervention successfully eliminated the decreased strength of preference.

#### Reconsideration Effect

The paired *t*-test was also used to check if prompting participants to reconsider their choice would yield different responses. There was no significant difference in the perceived source credibility in the first found (*M* = 48.73, *SD* = 20.27) and second round rating (*M* = 48.37, *SD* = 20.44) in the early intervention group, *t*(119) = .42, *p* = .672, *d* = .02, BF_01_ = 9.09. The Bayes factor provides moderate support for the null hypothesis. Similarly, there was no significant difference in the strength of preference between the first round (*M* = 39.89%, *SD* = 13.64) and second round ratings (*M* = 39.34%, *SD* = 14.47) in the early intervention group, *t*(119) = .75, *p* = .456, *d* = .04, BF_01_ = 7.69. The Bayes factor provides moderate support for the null hypothesis. These results indicate that asking participants to reconsider their choice had no effect on their responses to perceived source credibility and strength of preference questions.

### Discussion

This experiment found that the intervention on perceived source credibility could weaken or eliminate the decreased strength of preference. Early intervention failed to equalize perceived source credibility in the conflicting information condition and to eliminate decreased strength of preference, while late intervention succeeded. This may be due to interference in processing both the source occupation cues and the conflicting information simultaneously. Previous studies have shown that the simultaneous processing of two cognitive tasks can decrease the task accuracy and increase the reaction time in each task (Herath et al., 2001; Klingberg, 1998; Leone et al., 2017). The interference between two simultaneous tasks could be attributed to the limited cognitive resources, such as attention (Herath et al., 2001; Wickens, 2014), or the need of activation of the same part of the cortex or neural network (Klingberg, 1998; Pashler, 1994). These findings suggest that the simultaneous presentation of two different kinds of cues might reduce the level of processing of both, which explains the low effectiveness of the early intervention on perceived source credibility. These findings indicate that the timing of intervention could be a crucial factor when addressing the negative outcomes associated with conflicting information.

## General Discussion

The experiments in this paper provide a full test of the framework of conflictive uncertainty. Experiment 1 revealed that participants rated lower source credibility and strength of preference in conflicting information conditions compared to ambiguous information conditions. Furthermore, a mediation relationship was identified among the type of information, perceived source credibility, and strength of preference. Experiment 2 extended this mediation model by introducing perceived uncertainty as a second mediator, demonstrating that the effect of decreased source credibility on decreased strength of preference was mediated by increased perceived uncertainty. This experiment also manipulated perceived source credibility to observe changes in perceived uncertainty and strength of preference, effectively ruling out the potential reverse causality. Meanwhile, Experiment 2 further explored causal heterogeneity within this mediation relationship, showing that 36% of participants did not follow this pathway. Finally, Experiment 3 demonstrated that decreased strength of preference could be weakened or eliminated when decreased source credibility was neutralized through intervention.

These findings support the role of decreased source credibility and increased perceived uncertainty in explaining negative outcomes associated with conflicting information. The results align with prior research on trust and source credibility (Ding et al., 2011; Leiserowitz et al., 2013; Nagler et al., 2022; Shi et al., 2023), uncertainty and ambiguity (Han et al., 2009; Li et al., 2020; Nagler, 2013, 2022), preferences and behavioral intentions (Carpenter et al., 2014; Kobayashi, 2018; Marshall & Comello, 2019), and conflict aversion (Cabantous, 2007; Cabantous et al., 2011; Pushkarskaya et al., 2010, 2015; Smithson, 1999; Smithson et al., 2019). Moreover, these findings also resonate with established dual-process frameworks such as the Elaboration Likelihood Model (ELM), which emphasize the role of source credibility as a cognitive shortcut in decision-making (Chaiken & Trope, 1999; Kahneman & Frederick, 2002; Petty & Cacioppo, 1986). Meanwhile, this study advances these frameworks by positioning perceived uncertainty as a central mechanism underlying people’s avoidance of options or information from conflicting sources. This perspective bridges credibility models with emerging conflict and uncertainty management theories (Gottschling et al., 2019; Han et al., 2009, 2018; Stadtler & Bromme, 2014), providing a theoretical basis for managing the psychological impact of conflicting information.

The findings have meaningful implications for policymakers, science communicators, and public health practitioners who aim to mitigate the negative consequences of conflicting information, particularly in domains such as health or science communication. When individuals are exposed to conflictive uncertainty, such as contradictory claims about the safety or effectiveness of a vaccine, they may not trust both sources, or experience uncertainty and ambiguity in decision-making. To counter these effects, strategic interventions that enhance source credibility and reduce perceived uncertainty can play a critical role in maintaining public engagement with important but controversial topics.

Several strategies can be employed to foster audience engagement with topics involving conflictive uncertainty. First, emphasizing the source’s expertise or institutional reputation has been shown to enhance perceived credibility and facilitate message acceptance, particularly when the audiences lack strong prior attitudes or domain-specific knowledge (Hocevar et al., 2017; Metzger et al., 2010). Second, highlighting a shared social identity between the communicator and the audience, such as emphasizing shared community membership, political affiliation, can foster trust and encourage people to see the information as truthful rather than biased (Lee et al., 2019). Third, ensuring transparency in motives, funding sources, or evidence can help audiences differentiate between honest disagreement and manipulative disinformation, thus reducing perceived interpersonal uncertainty and eliminating aversion (Gehrau et al., 2021; Giubilini et al., 2023). The core of these strategies is to reduce negative attributions driven by suspicion, such as assuming bias, hidden agendas, or manipulation from the source, in the face of conflicting information. By addressing interpersonal uncertainty and improving the perceived trustworthiness and expertise of communicators, such interventions can redirect the public’s attention to the inherent epistemic limitations of the information. Ultimately, the public’s awareness of the uncertain nature of scientific findings could promote an informed, reflective public engagement in an increasingly complex and uncertain information environment.

Two remaining methodological and theoretical challenges should be highlighted, and future studies should aim to address them. First, there is still a lack of comprehensive individual-level measures of attitudes toward conflicting information and conflictive uncertainty. In terms of behavioral measures, Zhu et al. (2025) supported the use of uncertainty preference measures, such as the matching probability, for capturing group-level preferences, but also indicated their limitations in terms of the poor convergent validity and test–retest reliability in assessing individual differences. Regarding questionnaire or scale-based measures, the Ambiguity Aversion in Medicine (AA-Med) scale (Han et al., 2009, 2018) provides a valuable tool for assessing individuals’ attitudes toward conflicting information in medical contexts, but its applicability to other domains remains limited. The lack of reliable individual-level measures hinders efforts to investigate the moderating roles of individual differences in epistemic factors (e.g., need for closure), uncertainty attitudes (e.g., tolerance of uncertainty), and conflict resolution strategies (e.g., motivated reasoning) in this process. Thus, this challenge should be addressed first in future studies.

Second, there is a need to better understand the temporal dynamics of how individuals process and resolve conflictive uncertainty. According to the Content-Source Integration model (Stadtler & Bromme, 2014), the processing of conflicting information involves three stages: conflict detection, conflict regulation, and conflict resolution. Mapping psychological responses, such as increased perceived uncertainty, onto these stages may provide insight into the cognitive mechanisms underlying how people react to and manage conflictive uncertainty. For example, increased perceived uncertainty is likely to occur during the conflict detection stage, when individuals recognize contradictions between sources. This uncertainty may act as a signal that triggers epistemic discomfort or a sense of ambiguity, prompting efforts to manage the conflict cognitively. To regulate this discomfort, individuals may engage in motivated reasoning, including selectively trusting one source while discrediting the other to reduce perceived uncertainty. This process is likely present in the conflict resolution stage, where the individual settles on some cues (e.g., identities of sources) and forms a preferred explanation for information conflict (Gottschling et al., 2019). To empirically examine these temporal dynamics, future research could employ methods such as think-aloud protocols or neuroimaging techniques (e.g., fMRI, ERP), which allow for tracing nuanced cognitive processes related to uncertainty, trust, and reasoning in real time. These methods can help disentangle the sequential stages in reactions to conflicting information and conflictive uncertainty.

Some limitations of this study should be noted. First, the measures of source credibility were limited in two aspects: (1) they did not differentiate between factors of source credibility, such as trustworthiness and expertise, and (2) they did not assess the perceived credibility of each source individually. The first limitation restricted the ability to explore the effects of conflicting information on specific components of source credibility, while the second limited the investigation of conflictive uncertainty’s impact on the credibility of each individual source. Previous research suggests that people might assign lower credibility to sources that contradict their beliefs (Knobloch-Westerwick et al., 2015; Metzger et al., 2010). They may distrust certain information from the outset, making it easier for them to take a side and avoid conflictive uncertainty (Smithson, 2022). Moreover, factors such as motivated reasoning and political identity may also lead participants to actively dismiss certain sources (Boyer, 2021). Future research should assess the perceived credibility of each source and explore how factors like motivated reasoning or prior beliefs influence these perceptions.

Second, all findings in this paper were based on U.S. and U.K. samples, potentially limiting their generalizability to other cultural contexts. For example, Choi and Nisbett (2000) found that people from East Asian countries are more likely to accept contradiction and inconsistency as part of reality compared to those from Western countries. Consequently, they may exhibit lower aversion to conflicting information. Future research should explore cultural differences in conflict aversion to extend the framework’s relevance.

## Conclusion

In conclusion, this paper establishes a framework of conflictive uncertainty to explain the negative outcomes associated with conflicting information. It highlights the role of decreased source credibility and increased perceived uncertainty in driving the aversion to conflicting information. These findings offer a valuable foundation for strategies aimed at mitigating the negative effects of conflicting information on the overall society’s well-being.

## Supplemental Material

sj-docx-1-psp-10.1177_01461672251386102 – Supplemental material for Conflictive Uncertainty: A Framework for Understanding the Aversion to Conflicting Information in Social Contexts Supplemental material, sj-docx-1-psp-10.1177_01461672251386102 for Conflictive Uncertainty: A Framework for Understanding the Aversion to Conflicting Information in Social Contexts by Guangyu Zhu, Yiyun Shou, Michael Smithson and Michael J. Platow in Personality and Social Psychology Bulletin

## Appendix A

Example of the credibility questions based on Hanimann et al. (2022):

Q1. How credible do you think . . .?
Q2. How trustworthy do you think . . .?
Q3. How competent do you think . . .?
Q4. How authentic do you think . . .?
Q5. How believable do you think . . .?

**Table A1.** Correlations Matrix Between Credibility Items.

	Q2	Q3	Q4	Q5
Q1	0.889	0.778	0.846	0.838
Q2	-	0.782	0.816	0.826
Q3	-	-	0.810	0.771
Q4	-	-	-	0.791

Note: Cronbach’s alpha among the items = .81.

## Appendix B

**Table B1:** Correlations Matrix Between Different Variables in Experiment 1.

	SC	SP
TP	0.004	0.035
SC	-	0.351***

Note: *: significance at .05 level, **: significance at .01 level; ***: significance at .001 level.

TP = Trust Propensity; SC = Source Credibility; SP = Strength of Preference.

**Table B2:** Correlations Matrix Between Different Variables in Experiment 2.

	SC	SP	PU
TP	0.226***	-0.059	0.127*
SC	-	0.330***	0.622***
SP	-	-	0.346***

Note: *: significance at .05 level, **: significance at .01 level; ***: significance at .001 level.

TP = Trust Propensity; SC = Source Credibility; SP = Strength of Preference; PU = Perceived Uncertainty.

## Notes

1. This medium effect size is based on previous studies on the relative aversion to conflictive uncertainty over ambiguity, such as Smithson (1999) and Smithson et al. (2019).

2. This experiment selected conflicting probability estimations as the source of conflict for three reasons: (1) probability is a fundamental concept applicable across a wide range of scenarios, which allows for cross-domain comparison; (2) conflicting probability estimation can induce a sense of conflictive uncertainty. Although one might argue that conflicting probability estimations allow for compromise and may not fully simulate a sense of conflict, Smithson and Campbell (2009) demonstrated that participants perceive conflict when faced with such estimations; and (3) conflict aversion is a well-documented phenomenon observed consistently across different types of information (Cabantous, 2007; Cabantous et al., 2011; Pushkarskaya et al., 2010, 2015; Smithson, 1999; Smithson et al., 2019), so findings based on conflicting probability information can be generalized to scenarios involving other types of information.

3. Condition order effects have been tested (see supplementary materials for details), and there was no significant influence on the main outcomes, except for a small interaction effect with information type on perceived uncertainty, which did not affect the overall interpretation of results.

## References

[bibr1-01461672251386102] Adriatico J. M. Cruz A. Tiong R. C. Racho-Sabugo C. R. (2022). An analysis on the impact of choice overload to consumer decision paralysis. Journal of Economics, Finance and Accounting Studies, 4(1), 55–75. 10.32996/jefas.2022.4.1.4

[bibr2-01461672251386102] Aggarwal D. Mohanty P. (2022). Influence of imprecise information on risk and ambiguity preferences: Experimental evidence. Managerial and Decision Economics, 43(4), 1025–1038. 10.1002/mde.3436

[bibr3-01461672251386102] Ahn J. Kahlor L. A. (2023). When experts offer conflicting information: A study of perceived ambiguity, information insufficiency, trustworthiness and risk information behaviors. Health Communication, 38(14), 3276–3286. 10.1080/10410236.2022.2146033 36404712

[bibr4-01461672251386102] Alarcon G. M. Lyons J. B. Christensen J. C. Bowers M. A. Klosterman S. L. Capiola A. (2018). The role of propensity to trust and the five factor model across the trust process. Journal of Research in Personality, 75, 69–82. 10.1016/j.jrp.2018.05.006

[bibr5-01461672251386102] Alós-Ferrer C. Garagnani M. (2021). Choice consistency and strength of preference. Economics Letters, 198, 109672. 10.1016/j.econlet.2020.109672

[bibr6-01461672251386102] Baillon A. Huang Z. Selim A. Wakker P. P. (2018). Measuring ambiguity attitudes for all (natural) events. Econometrica, 86(5), 1839–1858. 10.3982/ecta14370

[bibr7-01461672251386102] Bates D. Maechler M. Bolker B. Walker S. Christensen R. H. B. Singmann H. Bolker M. B. (2015). Package “lme4.” Convergence, 12(1), 2. 10.32614/cran.package.lme4

[bibr8-01461672251386102] Bhanji J. P. Delgado M. R. (2014). The social brain and reward: Social information processing in the human striatum. Wiley Interdisciplinary Reviews: Cognitive Science, 5(1), 61–73. 10.1002/wcs.1266 PMC3890330 24436728

[bibr9-01461672251386102] Breidert C. Hahsler M. Reutterer T. (2006). A review of methods for measuring willingness-to-pay. Innovative Marketing, 2(4). 10.1007/s11002-006-5147-x

[bibr10-01461672251386102] Boyer M. M. (2021). Aroused argumentation: How the news exacerbates motivated reasoning. The International Journal of Press/Politics, 28(1), 92–115. 10.1177/19401612211010577

[bibr11-01461672251386102] Cabantous L. (2007). Ambiguity aversion in the field of insurance: Insurers’ attitude to imprecise and conflicting probability estimates. Theory and Decision, 62(3), 219–240. 10.1007/s11238-006-9015-1

[bibr12-01461672251386102] Cabantous L. Hilton D. Kunreuther H. Michel-Kerjan E. (2011). Is imprecise knowledge better than conflicting expertise? Evidence from insurers’ decisions in the United States. Journal of Risk and Uncertainty, 42, 211–232. 10.1007/s11166-011-9117-1

[bibr13-01461672251386102] Carpenter D. M. Elstad E. A. Blalock S. J. DeVellis R. F. (2014). Conflicting medication information: Prevalence, sources, and relationship to medication adherence. Journal of Health Communication, 19(1), 67–81. 10.1080/10810730.2013.798380 PMC8989251 24015878

[bibr14-01461672251386102] Carpenter D. M. Geryk L. L. Chen A. T. Nagler R. H. Dieckmann N. F. Han P. K. (2016). Conflicting health information: A critical research need. Health Expectations, 19(6), 1173–1182. 10.1111/hex.12438 PMC5139056 26709206

[bibr15-01461672251386102] Cavagnaro D. R. Regenwetter M. (2023). Probabilistic choice induced by strength of preference. Computational Brain & Behavior, 6(4), 569–600. 10.1007/s42113-023-00176-3

[bibr16-01461672251386102] Chaiken S. Trope Y. (Eds.). (1999). Dual-process theories in social psychology. Guilford Press.

[bibr17-01461672251386102] Choi I. Nisbett R. E. (2000). Cultural psychology of surprise: Holistic theories and recognition of contradiction. Journal of Personality and Social Psychology, 79(6), 890. 10.1037//0022-3514.79.6.890 11138759

[bibr18-01461672251386102] Clark D. Nagler R. H. Niederdeppe J. (2019). Confusion and nutritional backlash from news media exposure to contradictory information about carbohydrates and dietary fats. Public Health Nutrition, 22(18), 3336–3348. 10.1017/s1368980019002866 PMC10260685 31587681

[bibr19-01461672251386102] Clemence M. Jackson C. (2022). Ipsos global trustworthiness index 2022. https://www.ipsos.com/sites/default/files/ct/news/documents/2022-07/Global%20trustworthiness%202022%20Report.pdf

[bibr20-01461672251386102] Colquitt J. A. Scott B. A. LePine J. A. (2007). Trust, trustworthiness, and trust propensity: A meta-analytic test of their unique relationships with risk taking and job performance. Journal of Applied Psychology, 92(4), 909. 10.1037/0021-9010.92.4.909 17638454

[bibr21-01461672251386102] Dieckmann N. F. Johnson B. B. Gregory R. Mayorga M. Han P. K. Slovic P. (2017). Public perceptions of expert disagreement: Bias and incompetence or a complex and random world? Public Understanding of Science, 26(3), 325–338. 10.1177/0963662515603271 26346339

[bibr22-01461672251386102] Dimmock S. G. Kouwenberg R. Wakker P. P. (2016). Ambiguity attitudes in a large representative sample. Management Science, 62(5), 1363–1380. 10.1287/mnsc.2015.2198

[bibr23-01461672251386102] Ding D. Maibach E. W. Zhao X. Roser-Renouf C. Leiserowitz A. (2011). Support for climate policy and societal action are linked to perceptions about scientific agreement. Nature Climate Change, 1(9), 462–466. 10.1038/nclimate1295

[bibr24-01461672251386102] Ellsberg D. (1961). Risk, ambiguity, and the savage axioms. Quarterly Journal of Economics, 75(4), 643–669. 10.2307/1884324

[bibr25-01461672251386102] Euston D. R. Gruber A. J. McNaughton B. L. (2012). The role of medial prefrontal cortex in memory and decision making. Neuron, 76(6), 1057–1070. 10.1016/j.neuron.2012.12.002 PMC3562704 23259943

[bibr26-01461672251386102] Faul F. Erdfelder E. Buchner A. Lang A. G. (2009). Statistical power analyses using G* Power 3.1: Tests for correlation and regression analyses. Behavior Research Methods, 41(4), 1149–1160. 10.3758/brm.41.4.1149 19897823

[bibr27-01461672251386102] Gehrau V. Fujarski S. Lorenz H. Schieb C. Blöbaum B. (2021). The impact of health information exposure and source credibility on COVID-19 vaccination intention in Germany. International Journal of Environmental Research and Public Health, 18(9), 4678. 10.3390/ijerph18094678 PMC8124400 33924796

[bibr28-01461672251386102] Gillman A. S. Scharnetzki L. Boyd P. Ferrer R. A. Klein W. M. Han P. K. (2023). Perceptions and tolerance of uncertainty: Relationship to trust in COVID-19 health information and vaccine hesitancy. Journal of Behavioral Medicine, 46(1), 40–53. 10.1007/s10865-022-00302-9 PMC8990605 35394240

[bibr29-01461672251386102] Giubilini A. Gur-Arie R. Jamrozik E. (2023). Expertise, disagreement, and trust in vaccine science and policy. The importance of transparency in a world of experts. Diametros, 27, 7–27. 10.33392/diam.1871 PMC7616594 39404699

[bibr30-01461672251386102] Gottschling S. Kammerer Y. Gerjets P. (2019). Readers’ processing and use of source information as a function of its usefulness to explain conflicting scientific claims. Discourse Processes, 56(5–6), 429–446. 10.1080/0163853x.2019.1610305

[bibr31-01461672251386102] Gugerty L. Link D. M. (2020). How heuristic credibility cues affect credibility judgments and decisions. Journal of Experimental Psychology: Applied, 26(4), 620. 10.1037/xap0000279 32658526

[bibr32-01461672251386102] Hämeen-Anttila K. Nordeng H. Kokki E. Jyrkkä J. Lupattelli A. Vainio K. Enlund H. (2014). Multiple information sources and consequences of conflicting information about medicine use during pregnancy: A multinational internet-based survey. Journal of Medical Internet Research, 16(2), e60. 10.2196/jmir.2939 PMC3961698 24565696

[bibr33-01461672251386102] Han P. K. Reeve B. B. Moser R. P. Klein W. M. (2009). Aversion to ambiguity regarding medical tests and treatments: Measurement, prevalence, and relationship to sociodemographic factors. Journal of Health Communication, 14(6), 556–572. 10.1080/10810730903089630 PMC4205160 19731127

[bibr34-01461672251386102] Han P. K. Zikmund-Fisher B. J. Duarte C. W. Knaus M. Black A. Scherer A. M. Fagerlin A. (2018). Communication of scientific uncertainty about a novel pandemic health threat: Ambiguity aversion and its mechanisms. Journal of Health Communication, 23(5), 435–444. 10.1080/10810730.2018.1461961 PMC6029253 29648962

[bibr35-01461672251386102] Hanimann A. Heimann A. Hellmueller L. Trilling D. (2022). Believing in credibility measures: Reviewing credibility measures in media research from 1951 to 2018. International Journal of Communication, 17, 22. https://ijoc.org/index.php/ijoc/article/view/18815

[bibr36-01461672251386102] Herath P. Klingberg T. Young J. Amunts K. Roland P. (2001). Neural correlates of dual task interference can be dissociated from those of divided attention: An fMRI study. Cerebral Cortex, 11(9), 796–805. 10.1093/cercor/11.9.796 11532885

[bibr37-01461672251386102] Heyns M. Rothmann S. (2015). Dimensionality of trust: An analysis of the relations between propensity, trustworthiness and trust. SA Journal of Industrial Psychology, 41(1), 1–12. 10.4102/sajip.v41i1.1263

[bibr38-01461672251386102] Hocevar K. P. Metzger M. Flanagin A. J. (2017). Source credibility, expertise, and trust in health and risk messaging. In Oxford research Encyclopedia of communication. Oxford University Press. 10.1093/acrefore/9780190228613.013.287

[bibr39-01461672251386102] Hothorn T. Bretz F. Westfall P. Heiberger R. M. Schuetzenmeister A. Scheibe S. Hothorn M. T. (2016). Package “multcomp.” Simultaneous inference in general parametric models. Project for Statistical Computing. 10.32614/cran.package.multcomp

[bibr40-01461672251386102] Hovland C. I. Janis I. L. Kelley H. H. (1953). Communication and persuasion. 10.1007/bf02713272

[bibr41-01461672251386102] Hsu M. Camerer C. F. (2004). Ambiguity-aversion in the Brain. Academy of Behavioral Finance & Economics. https://citeseerx.ist.psu.edu/document?repid=rep1&type=pdf&doi=d9d72a46cfa9de4a7fcccb14bb3982efb1163681

[bibr42-01461672251386102] Hu Y. Shyam Sundar S. (2010). Effects of online health sources on credibility and behavioral intentions. Communication Research, 37(1), 105–132. 10.1177/0093650209351512

[bibr43-01461672251386102] Iles I. A. Gillman A. S. O’Connor L. E. Ferrer R. A. Klein W. M. P. (2022). Understanding responses to different types of conflicting information about cancer prevention. Social Science & Medicine, 311, 115292. 10.1016/j.socscimed.2022.115292 36063595

[bibr44-01461672251386102] Imai K. Keele L. Tingley D. (2010). A general approach to causal mediation analysis. Psychological Methods, 15(4), 309. 10.1037/a0020761 20954780

[bibr45-01461672251386102] Imai K. Keele L. Yamamoto T. (2010). Identification, inference and sensitivity analysis for causal mediation effects. Statistical Science, 25(1), 51. 10.1214/10-sts321

[bibr46-01461672251386102] Jasielska D. Rogoza R. Zajenkowska A. Russa M. B. (2021). General trust scale: Validation in cross-cultural settings. Current Psychology, 40, 5019–5029. 10.1007/s12144-019-00435-2

[bibr47-01461672251386102] Jensen J. D. Hurley R. J. (2012). Conflicting stories about public scientific controversies: Effects of news convergence and divergence on scientists’ credibility. Public Understanding of Science, 21(6), 689–704. 10.1177/0963662510387759 23832155

[bibr48-01461672251386102] Kahneman D. Frederick S. (2002). Representativeness revisited: Attribute substitution in intuitive judgment. In Heuristics and biases (pp. 49–81). 10.1017/cbo9780511808098.004

[bibr49-01461672251386102] Klingberg T. (1998). Concurrent performance of two working memory tasks: Potential mechanisms of interference. Cerebral Cortex, 8(7), 593–601. 10.1093/cercor/8.7.593 9823480

[bibr50-01461672251386102] Knobloch-Westerwick S. Johnson B. K. Silver N. A. Westerwick A. (2015). Science exemplars in the eye of the beholder. Science Communication, 37(5), 575–601. 10.1177/1075547015596367

[bibr51-01461672251386102] Kobayashi K. (2018). Effects of conflicting scientific arguments on belief change: Argument evaluation and expert consensus perception as mediators. Journal of Applied Social Psychology, 48(4), 177–187. 10.1111/jasp.12499

[bibr52-01461672251386102] Köbberling V. (2006). Strength of preference and cardinal utility. Economic Theory, 27, 375–391. 10.1007/s00199-005-0598-5

[bibr53-01461672251386102] Kocher M. G. Lahno A. M. Trautmann S. T. (2018). Ambiguity aversion is not universal. European Economic Review, 101, 268–283. 10.1016/j.euroecorev.2017.09.016

[bibr54-01461672251386102] Koehler J. J. Gershoff A. D. (2003). Betrayal aversion: When agents of protection become agents of harm. Organizational Behavior and Human Decision Processes, 90(2), 244–261. 10.1016/s0749-5978(02)00518-6

[bibr55-01461672251386102] Krahnen J. P. Rieck C. Theissen E. (1997). Inferring risk preferences from certainty equivalents: Some lessons from an experimental study. Journal of Economic Psychology, 18(5), 469–486. 10.1016/s0167-4870(97)00019-6

[bibr56-01461672251386102] Lee C. Nagler R. H. Wang N. (2017). Source-specific exposure to contradictory nutrition information: Documenting prevalence and effects on adverse cognitive and behavioral Outcomes. Health Communication, 33(4), 453–461. 10.1080/10410236.2016.1278495 PMC6102724 28151010

[bibr57-01461672251386102] Lee G. C. Platow M. J. Augoustinos M. Van Rooy D. Spears R. Bar Tal D. (2019). When are anti-fat attitudes understood as prejudice vs. truth? An experimental study of social influence effects. Obesity Science & Practice, 5, 28–35. 10.1002/osp4.315 PMC6381298 30820328

[bibr58-01461672251386102] Leiserowitz A. A. Maibach E. W. Roser-Renouf C. Smith N. Dawson E. (2013). Climategate, public opinion, and the loss of trust. American Behavioral Scientist, 57(6), 818–837. 10.1177/0002764212458272

[bibr59-01461672251386102] Leone C. Feys P. Moumdjian L. D’Amico E. Zappia M. Patti F. (2017). Cognitive-motor dual-task interference: A systematic review of neural correlates. Neuroscience & Biobehavioral Reviews, 75, 348–360. 10.1016/j.neubiorev.2017.01.010 28104413

[bibr60-01461672251386102] Lewicki R. J. Bunker B. B. (1996). Developing and maintaining trust in work relationships. Trust in Organizations: Frontiers of Theory and Research, 114(139), 30. 10.4135/9781452243610.n7

[bibr61-01461672251386102] Lewis D. (1973). Counterfactuals and comparative possibility. In Harper W. L. Stalnaker R. Pearce G. (Eds.), IFS: Conditionals, belief, decision, chance and time (pp. 57–85). Springer. 10.1007/978-94-009-9117-0_3

[bibr62-01461672251386102] Li J. Y. Wen J. Kim J. McKeever R. (2020). Applying the theory of motivated information management to the context of conflicting online health information: Implications for childhood vaccination communication with parents. International Journal of Strategic Communication, 14(5), 330–347. 10.1080/1553118x.2020.1817030

[bibr63-01461672251386102] Liu H. H. Chang J. H. (2017). Relationship type, perceived trust, and ambiguity aversion. Marketing Letters, 28, 255–266. 10.1007/s11002-016-9408-z

[bibr64-01461672251386102] Loeys T. Moerkerke B. Vansteelandt S. (2015). A cautionary note on the power of the test for the indirect effect in mediation analysis. Frontiers in Psychology, 5, 1549. 10.3389/fpsyg.2014.01549 PMC4290592 25628585

[bibr65-01461672251386102] Lucassen T. Schraagen J. M. (2012). Propensity to trust and the influence of source and medium cues in credibility evaluation. Journal of Information Science, 38(6), 566–577. 10.1177/0165551512459921

[bibr66-01461672251386102] Machina M. J. Siniscalchi M. (2014). Ambiguity and ambiguity aversion. In Handbook of the Economics of Risk and Uncertainty (pp. 729–807). 10.1016/b978-0-444-53685-3.00013-1

[bibr67-01461672251386102] Marshall L. H. Comello M. L. (2019). Stymied by a wealth of health information: How viewing conflicting information online diminishes efficacy. Journal of Communication in Healthcare, 12(1), 4–12. 10.1080/17538068.2019.1580064

[bibr68-01461672251386102] Metzger M. J. Flanagin A. J. Medders R. B. (2010). Social and heuristic approaches to credibility evaluation online. Journal of Communication, 60(3), 413–439. 10.1111/j.1460-2466.2010.01488.x

[bibr69-01461672251386102] Morey R. D. Rouder J. N. Jamil T. Morey M. R. D. (2015). Package “bayesfactor.” 10.32614/cran.package.bayesfactor

[bibr70-01461672251386102] Nagler R. H. (2013). Adverse outcomes associated with media exposure to contradictory nutrition messages. Journal of Health Communication, 19(1), 24–40. 10.1080/10810730.2013.798384 PMC4353569 24117281

[bibr71-01461672251386102] Nagler R. H. Vogel R. I. Gollust S. E. Yzer M. C. Rothman A. J. (2021). Effects of prior exposure to conflicting health information on responses to subsequent unrelated health messages: Results from a population-based longitudinal experiment. Annals of Behavioral Medicine, 56(5), 498–511. 10.1093/abm/kaab069 PMC9116588 34398961

[bibr72-01461672251386102] Nagler R. H. Gollust S. E. Yzer M. C. Vogel R. I. Rothman A. J. (2023). Sustaining positive perceptions of science in the face of conflicting health information: An experimental test of messages about the process of scientific discovery. Social Science & Medicine, 334, 116194. 10.1016/j.socscimed.2023.116194 PMC10552003 37660521

[bibr73-01461672251386102] Nagler R. H. LoRusso S. M. (2017). Conflicting information and message competition in health and risk messaging. In Oxford research Encyclopedia of communication. 10.1093/acrefore/9780190228613.013.292

[bibr74-01461672251386102] Nagler R. H. Vogel R. I. Gollust S. E. Rothman A. J. Fowler E. F. Yzer M. C. (2020). Public perceptions of conflicting information surrounding COVID-19: Results from a nationally representative survey of U.S. adults. PLoS One, 15(10), e0240776. 10.1371/journal.pone.0240776 PMC7577476 33085719

[bibr75-01461672251386102] Nagler R. H. Vogel R. I. Gollust S. E. Yzer M. C. Rothman A. J. (2022). Effects of prior exposure to conflicting health information on responses to subsequent unrelated health messages: Results from a population-based longitudinal experiment. Annals of Behavioral Medicine, 56(5), 498–511. 10.1093/abm/kaab069 PMC9116588 34398961

[bibr76-01461672251386102] Nagler R. H. Yzer M. C. Rothman A. J. (2019). Effects of media exposure to conflicting information about mammography: Results from a population-based survey experiment. Annals of Behavioral Medicine, 53(10), 896–908. 10.1093/abm/kay098 PMC6735717 30596830

[bibr77-01461672251386102] Niederdeppe J. Levy A. G. (2007). Fatalistic beliefs about cancer prevention and three prevention behaviors. Cancer Epidemiology, Biomarkers & Prevention, 16(5), 998–1003. 10.1158/1055-9965.epi-06-0608 17507628

[bibr78-01461672251386102] Pashler H. (1994). Dual-task interference in simple tasks: Data and theory. Psychological Bulletin, 116(2), 220. 10.1037//0033-2909.116.2.220 7972591

[bibr79-01461672251386102] Pearl J. (2009). Causality. Cambridge university press. 10.1017/cbo9780511803161

[bibr80-01461672251386102] Petty R. E. Cacioppo J. T. (1986). The elaboration likelihood model of persuasion. In Communication and Persuasion (pp. 1–24). 10.1007/978-1-4612-4964-1_1

[bibr81-01461672251386102] Pushkarskaya H. Liu X. Smithson M. Joseph J. E. (2010). Beyond risk and ambiguity: Deciding under ignorance. Cognitive, Affective, & Behavioral Neuroscience, 10, 382–391. 10.3758/cabn.10.3.382 20805539

[bibr82-01461672251386102] Pushkarskaya H. Smithson M. Joseph J. E. Corbly C. Levy I. (2015). Neural correlates of decision-making under ambiguity and conflict. Frontiers in Behavioral Neuroscience, 9, 325. 10.3389/fnbeh.2015.00325 PMC4661279 26640434

[bibr83-01461672251386102] Rosseel Y. Oberski D. Byrnes J. Vanbrabant L. Savalei V. Merkle E. Jorgensen T. (2017). Package “lavaan.” 10.32614/cran.package.lavaan

[bibr84-01461672251386102] Roster C. A. Lucianetti L. Albaum G. (2015). Exploring slider vs. categorical response formats in web-based surveys. Journal of Research Practice, 11(1). http://jrp.icaap.org/index.php/jrp/rt/printerFriendly/509/413

[bibr85-01461672251386102] Rubin D. B. (2005). Causal inference using potential outcomes. Journal of the American Statistical Association, 100(469), 322–331. 10.1198/016214504000001880

[bibr86-01461672251386102] Schoemann A. M. Boulton A. J. Short S. D. (2017). Determining power and sample size for simple and complex mediation models. Social Psychological and Personality Science, 8(4), 379–386. 10.1177/1948550617715068

[bibr87-01461672251386102] Schweitzer M. E. Hershey J. C. Bradlow E. T. (2006). Promises and lies: Restoring violated trust. Organizational Behavior and Human Decision Processes, 101(1), 1–19. 10.1016/j.obhdp.2006.05.005

[bibr88-01461672251386102] Shi W. Rothman A. J. Yzer M. C. Nagler R. H. (2023). Effects of exposure to conflicting information about mammography on cancer information overload, perceived scientists’ credibility, and perceived journalists’ credibility. Health Communication, 38(11), 2481–2490. 10.1080/10410236.2022.2077163 PMC9681936 35607276

[bibr89-01461672251386102] Sim M. Kim S. Y. Suh Y. (2022). Sample size requirements for simple and complex mediation models. Educational and Psychological Measurement, 82(1), 76–106. 10.1177/00131644211003261 PMC8725051 34992307

[bibr90-01461672251386102] Smithson M. (1999). Conflict aversion: Preference for ambiguity vs. conflict in sources and evidence. Organizational Behavior and Human Decision Processes, 79(3), 179–198. 10.1006/obhd.1999.2844 10471360

[bibr91-01461672251386102] Smithson M. (2022). The psychology of conflictive uncertainty. In Recent advancements in multi-view data analytics (pp. 1–21). Springer. 10.1007/978-3-030-95239-6_1

[bibr92-01461672251386102] Smithson M. Campbell P. D. (2009). Buying and selling prices under risk, ambiguity and conflict. In 6th international symposium on imprecise probability: Theories and applications.

[bibr93-01461672251386102] Smithson M. Priest D. Shou Y. Newell B. R. (2019). Ambiguity and conflict aversion when uncertainty is in the outcomes. Frontiers in Psychology, 10, 539. 10.3389/fpsyg.2019.00539 PMC6450420 30984054

[bibr94-01461672251386102] Sobel M. E. (1982). Asymptotic confidence intervals for indirect effects in structural equation models. Sociological Methodology, 13, 290–312. 10.2307/270723

[bibr95-01461672251386102] Stadtler M. Bromme R. (2014). The content–source integration model: A taxonomic description of how readers comprehend conflicting scientific information. In Processing Inaccurate Information (pp. 379–402). 10.7551/mitpress/9737.003.0023

[bibr96-01461672251386102] Tan A. S. L. Lee C. Nagler R. H. Bigman C. A. (2017). To vape or not to vape? Effects of exposure to conflicting news headlines on beliefs about harms and benefits of electronic cigarette use: Results from a randomized controlled experiment. Preventive Medicine, 105, 97–103. 10.1016/j.ypmed.2017.08.024 PMC5653463 28882742

[bibr97-01461672251386102] Taplin S. H. Urban N. Taylor V. M. Savarino J. (1997). Conflicting national recommendations and the use of screening mammography: Does the physician’s recommendation matter?. The Journal of the American Board of Family Practice, 10(2), 88–95. 10.3122/jabfm.10.2.88 9071688

[bibr98-01461672251386102] Thielmann I. Hilbig B. E. (2015). Trust: An integrative review from a person–situation perspective. Review of General Psychology, 19(3), 249–277. 10.1037/gpr0000046

[bibr99-01461672251386102] Thielmann I. Spadaro G. Balliet D. (2020). Personality and prosocial behavior: A theoretical framework and meta-analysis. Psychological Bulletin, 146(1), 30. 10.1037/bul0000217 31841013

[bibr100-01461672251386102] Tingley D. Yamamoto T. Hirose K. Keele L. Imai K. (2014). Mediation: R package for causal mediation analysis. 10.18637/jss.v059.i05

[bibr101-01461672251386102] Visschers V. H. (2017). Judgments under uncertainty: Evaluations of univocal, ambiguous and conflicting probability information. Journal of Risk Research, 20(2), 237–255. 10.1080/13669877.2015.1043569

[bibr102-01461672251386102] Weyerer J. C. Wirtz B. W. Thomas M. J. (2019). Influencing factors of online reviews: An empirical analysis of determinants of purchase intention. International Journal of Electronic Business, 15(1), 43. 10.1504/ijeb.2019.10020274

[bibr103-01461672251386102] Wickens C. D. (2014). The structure of attentional resources. In Nickerson R. S. (Ed.), Attention and performance VIII (pp. 239–257). Psychology Press. 10.4324/9781315802961

[bibr104-01461672251386102] Yamagishi T. Yamagishi M. (1994). Trust and commitment in the United States and Japan. Motivation and Emotion, 18(2), 129–166. 10.1007/bf02249397

[bibr105-01461672251386102] Zhu G. Shou Y. Smithson M. (2025). An investigation of the convergent validity and test–retest reliability of three uncertainty preference measures. Behavior Research Methods, 57(8). 10.3758/s13428-025-02729-9 PMC12241273 40634814

