# Supplementary material for: Conflictive Uncertainty: A Framework for Understanding the Aversion to Conflicting Information in Social Contexts
Source: Pers Soc Psychol Bull. 2025 Nov 20;52(11):3844–61. doi: 10.1177/01461672251386102 (PMC13646403; doi:10.1177/01461672251386102)
Supplement: Supplementary file 1 [file sj-docx-1-psp-10.1177_01461672251386102.docx]

Supplementary Materials – Replication of Experiment 2

Experiment 2b

Method

Participants and Design

A total of 393 English-speaking adult participants from the USA and UK participated in this experiment. This experiment is a between-subject design, with both type of information (ambiguous information vs. conflicting information) and occupation of the sources (doctor vs. politician vs. no occupation information) as between-subject factors. Participants were randomly assigned to either ambiguous or conflicting information condition. One third of them received information from doctors, one third of them received information from politicians, and the rest were not given cues on source occupation. Based on the G*power 3.1 (Faul et al., 2009), the sample size of 393 was sufficient to detect a medium main effect (η^2^ = 0.06) with 0.05 significance level and 0.90 power in between-subject ANOVA. This sample size is also sufficient to detect a medium size sequential mediation relationship with 0.80 power (Schoemann et al., 2017).

The participants had a mean age of 39.21 (*SD* = 15.03). The sample consisted of 45% males, 53% females, and 2% non-binary gender. Participants included 81% Caucasians, 6% African, 8% Asians, and 5% people with mixed racial backgrounds. Participants were recruited through an online platform Prolific. They were paid £0.6 (around US$0.78) for their participation.

All data, analysis code, and research materials are available at https://osf.io/tcv63. Data were analysed using R, version 4.3.1. The preregistration can be found at https://osf.io/y6n7e. Hypothesis 2 was preregistered. Hypothesis 1, 2, 3, 4, 5 was registered. However, the preregistered beta regression analysis was replaced with a between-subjects ANOVA to maintain consistency with previous analyses. A comparison of the results from the beta regression and the between-subjects ANOVA revealed no instances where one model produced significant results while the other did not.

Measures

This experiment selected the health scenario from Experiment 1. The trust propensity question, perceived source credibility question, and strength of preference were the same as the health scenario in Experiment 1. There were additional cues on source occupations below each source’s name.

**Perceived uncertainty.** Perceived uncertainty was measured in a slider question from 0 (“not certain at all”) to 100 (“very certain”). Participants were asked, asking: “How certain do you feel about effectiveness of the medicine?”.

***Procedure***

The procedure of Experiment 2b was similar to Experiment 1.

Results

The descriptive statistics of participants’ trust propensity, perceived source credibility, perceived uncertainty, and strength of preference are summarised in Table 1. This table also includes the effect size (Cohen’s *d*) of the difference in these variables between the ambiguous and conflicting information conditions.

**Table 1**
Description information of the variables

|  | Ambiguous information | | | Conflicting information | | | Cohen’s *d* |
| --- | --- | --- | --- | --- | --- | --- | --- |
| Variables | *Mean* | *Median* | *SD* | *Mean* | *Median* | *SD* |  |
| Control | | | | | | | |
| TP | 3.46 | 3.50 | 0.65 | 3.44 | 3.50 | 0.68 | 0.03 |
| SC | 52.69 | 57.00 | 23.55 | 37.18 | 40.00 | 20.40 | 0.70 |
| PU | 16.58 | 50.00 | 24.05 | 34.63 | 35.00 | 19.44 | 0.83 |
| SP | 44.27% | 47.50% | 15.45 | 39.87% | 40.00% | 14.75 | 0.29 |
| Doctor | | | | | | | |
| TP | 3.52 | 3.50 | 0.61 | 3.51 | 3.50 | 0.62 | 0.02 |
| SC | 65.23 | 70.00 | 23.59 | 53.41 | 50.00 | 19.73 | 0.54 |
| PU | 52.90 | 55.00 | 21.27 | 41.12 | 45.00 | 20.59 | 0.56 |
| SP | 46.54% | 50.00% | 9.95 | 42.16% | 47.50% | 11.34 | 0.41 |
| Politician | | | | | | | |
| TP | 3.59 | 3.67 | 0.52 | 3.46 | 3.50 | 0.67 | 0.22 |
| SC | 36.98 | 37.00 | 23.66 | 27.56 | 23.50 | 20.40 | 0.43 |
| PU | 39.82 | 40.00 | 20.67 | 26.65 | 27.00 | 19.84 | 0.65 |
| SP | 35.83% | 40.00% | 14.16 | 34.96% | 36.25% | 19.41 | 0.05 |

Note: TP = Trust Propensity; SC = Source Credibility; PU = Perceived uncertainty; SP = Strength of Preference

**Perceived Source Credibility.** A 2 (type of information: ambiguous vs. conflicting) * 3 (source occupation type: control vs. doctor vs. politician) between-subject ANOVA was conducted on perceived source credibility. The result found a significant main effect of information type on the perceived source credibility, *F*(1, 387) = 40.18, *p* < .001, η^2^ = .094. Participants reported a higher perceived credibility for sources providing ambiguous information (*M* = 52.55, *SD* = 26.20) compared to those providing conflicting information (*M* = 38.52, *SD* = 22.69), supporting Hypothesis 1. The source occupation also had a significant main effect on the perceived source credibility, *F*(2, 387) = 49.31, *p* < .001, η^2^ =.203. The post-hoc Tukey contrasts test showed that there was a significant lower perceived source credibility in the control condition (*M* = 44.64, *SD* = 23.22) than in the doctor condition (*M* = 59.95, *SD* = 22.65), *z* = 3.79, *p* = .001 There was also a significant higher perceived source credibility in the control condition (*M* = 44.64, *SD* = 23.22) than in the politician condition (*M* = 31.84, *SD* = 22.36), *z* = 3.95, *p* < .001. The difference in perceived source credibility between doctor condition and politician condition was also significant, *z* = 3.82, *p* < .001. The interaction between type of information and source occupation type was not significant (*p* = .532).

**Perceived uncertainty.** A between-subject ANOVA found a significant main effect of information type on the perceived uncertainty, *F*(1, 387) = 38.87, *p* < .001, η^2^ = .092. Participants reported a lower level of uncertainty in the ambiguous information condition (*M* = 46.87, *SD* = 22.57) compared to the conflicting information condition (*M* = 33.66, *SD* = 20.69), supporting Hypothesis 2. The source occupation also had a significant main effect on the perceived uncertainty, *F*(2, 387) = 14.17, *p* < .001, η^2^ =.073, supporting Hypothesis 4. The post-hoc Tukey contrasts test showed that there was a significantly lower perceived uncertainty in the doctor condition (*M* = 47.64, *SD* = 21.70), compared to in the politician condition (*M* = 32.63, *SD* = 21.19), *z* = 3.57, *p* = .001. The interaction between type of information and source occupation type was not significant (*p* = .959).

**Strength of Preference.** A between-subject ANOVA found a significant main effect of information type on the strength of preference, *F*(1, 387) = 6.55, *p* = .011, η^2^ = .017. Participants demonstrated a higher strength of preference for options with ambiguous information (*M* = 42.53, *SD* = 13.92) compared to those with conflicting information (*M* = 38.77, *SD* = 15.99), supporting Hypothesis 3. The source occupation also had a significant main effect on the strength of preference, *F*(2, 387) = 13.18, *p* < .001, η^2^ =.064, supporting Hypothesis 5. The post-hoc Tukey contrasts test showed that there was no significant difference in strength of preference between the control condition (*M* = 41.98, *SD* = 15.19) and the doctor condition (*M* = 44.58, *SD* = 10.78), *z* = 0.90, *p* = .368, but there was a significant higher strength of preference in the control condition (*M* = 41.98, *SD* = 15.19) than in the politician condition (*M* = 35.36, *SD* = 17.16), *z* = 3.20, *p* = .003. The interaction between type of information and source occupation type was not significant (*p* = .530).

**Sequential Mediation Analysis.** A sequential mediation model (using the mediation package; Rosseel et al., 2017) was conducted to examine the relationships among type of information, perceived source credibility, source occupation, perceived uncertainty, and strength of preference. In the type of information variable, the ambiguous information condition was coded as 0, and the conflicting information condition was coded as 1. For the source occupation variable, the doctor condition was coded as 1, the control condition as 0, and the politician condition as -1. The results of the sequential mediation model are illustrated in Figure 1.

The whole model showed a good fit, χ²(1) = 8.69, *p* = .003, CFI = .982, RMSEA = .140, SRMR = .028. There was a non-significant total effect of type of information on strength of preference, β = -2.45, 95% CI = [-5.29, 0.39], *p* = .092. However, there was a significant indirect effect (X-M_1_-M_2_-Y) from type of information, via perceived source credibility, perceived uncertainty, to strength of preference, β = -1.01, 95% CI = [-1.67, -0.34], *p* = .003. There was also a significant indirect effect (X-M_2_-Y) from type of information, vis perceived uncertainty, to strength of preference, β = -0.92, 95% CI = [-1.39, -0.05], *p* = .034. However, there was a non-significant indirect effect (X-M_1_-Y) from type of information via perceived source credibility to strength of preference, β = -0.88, 95% CI = [-1.96, 0.19], *p* = .113.

**Figure 1**

*Sequential Mediation Models Results*

Note: *: significance at 0.05 level, **: significance at 0.01 level; ***: significance at 0.001 level

**Table 2**

*Correlations matrix between different variables in Experiment 2b*

|  | SC | SP | PU |
| --- | --- | --- | --- |
| TP | 0.096 | 0.008 | 0.214** |
| SC | - | 0.362*** | 0.673*** |
| SP | - | - | 0.335*** |

Note: *: significance at 0.05 level, **: significance at 0.01 level; ***: significance at 0.001 level; TP = Trust Propensity; SC = Source Credibility; SP = Strength of Preference; PU = Perceived Uncertainty

**Supplementary Materials - Effect of Condition Order on Main Outcomes**

To assess whether the order in which participants encountered ambiguous versus conflicting information influenced their responses, we conducted additional analyses including condition order (ambiguous-first vs. conflicting-first) as a between-subjects factor in the models and tested its interaction with other main effects.

In the model with information type (TP) and source occupation (SO) as independent variable, and perceived source credibility as outcome variable, it found no significant main effect of condition order (F(1, 189) = 0.80, p = .372), and no significant interactions involving order (TP * Order: F(1, 191) = 0.59, p = .443; SO * Order: F(2, 189) = 0.22, p = .804).

In the model with information type (TP) and source occupation (SO) as independent variables and perceived uncertainty as the outcome variable, there was no significant main effect of condition order, F(1, 189) = 0.33, p = .564, and no significant interactions (SO * Order: F(2, 189) = 0.42, p = .656; TP * SO * Order: F(2, 189) = 0.48, p = .622). However, there was a small significant interaction between information type and condition order, F(1, 189) = 6.10, p = .014, partial η² = 0.03. Follow-up pairwise comparisons with Holm adjustment showed a larger difference in perceived uncertainty when ambiguous information appeared first (diff = 15.35, t(191) = 7.48, d = 0.54, p < .001) than when conflict information appeared first (diff = 8.04, t(191) = 3.78, d = 0.27, p < .001). A plausible explanation may be expectancy violation: starting with ambiguous information sets an expectation of coherence, and conflict information presented second violates that expectation, increasing uncertainty.

In the model with information type (TP) and source occupation (SO) as independent variables and strength of preference as the outcome variable, there was no significant main effect of condition order, F(1, 189) = 3.49, p = .063, and no significant interactions involving order (TP * Order: F(1, 189) = 0.09, p = .768; SO * Order: F(2, 189) = 0.16, p = .850; TP * SO * Order: F(2, 189) = 0.75, p = .474).

These results suggest that condition order have minimal effect on the main outcomes of our study.
